# Alzheimer’s disease risk allele of *PICALM* causes detrimental lipid droplets in microglia

**DOI:** 10.21203/rs.3.rs-4407146/v1

**Published:** 2024-05-24

**Authors:** Alena Kozlova, Siwei Zhang, Ari Sudwarts, Hanwen Zhang, Stanislau Smirnou, Xiaotong Sun, Kimberly Stephenson, Xiaojie Zhao, Brendan Jamison, Moorthi Ponnusamy, Xin He, Zhiping P. Pang, Alan R. Sanders, Hugo J. Bellen, Gopal Thinakaran, Jubao Duan

**Affiliations:** 1 Center for Psychiatric Genetics, NorthShore University HealthSystem, Evanston, IL 60201, USA.; 2 Byrd Alzheimer’s Center and Research Institute, University of South Florida, Tampa, FL 33613, USA.; 3 Department of Molecular Medicine, Morsani College of Medicine, University of South Florida, Tampa, FL 33612, USA.; 4 Department of Human Genetics, The University of Chicago, Chicago, IL 60637, USA.; 5 Department of Neuroscience and Cell Biology, Child Health Institute of New Jersey, Rutgers Robert Wood Johnson Medical School, New Brunswick, NJ 08901, USA.; 6 Grossman Institute for Neuroscience, Quantitative Biology and Human Behavior, The University of Chicago, Chicago, IL 60637, USA.; 7 Department of Psychiatry and Behavioral Neuroscience, The University of Chicago, Chicago, IL 60637, USA.; 8 Department of Molecular and Human Genetics, Baylor College of Medicine, Houston, TX 77030, USA.; 9 Jan and Dan Duncan Neurological Research Institute, Texas Children’s Hospital, Houston, TX 77030, USA.; 10 Department of Neuroscience, Baylor College of Medicine, Houston, TX 77030, USA.

## Abstract

Despite genome-wide association studies of late-onset Alzheimer’s disease (LOAD) having identified many genetic risk loci^1–6^, the underlying disease mechanisms remain largely unknown. Determining causal disease variants and their LOAD-relevant cellular phenotypes has been a challenge. Leveraging our approach for identifying functional GWAS risk variants showing allele-specific open chromatin (ASoC)^7^, we systematically identified putative causal LOAD risk variants in human induced pluripotent stem cells (iPSC)-derived neurons, astrocytes, and microglia (MG) and linked *PICALM* risk allele to a previously unappreciated MG-specific role of *PICALM* in lipid droplet (LD) accumulation. ASoC mapping uncovered functional risk variants for 26 LOAD risk loci, mostly MG-specific. At the MG-specific *PICALM* locus, the LOAD risk allele of rs10792832 reduced transcription factor (PU.1) binding and *PICALM* expression, impairing the uptake of amyloid beta (Aβ) and myelin debris. Interestingly, MG with *PICALM* risk allele showed transcriptional enrichment of pathways for cholesterol synthesis and LD formation. Genetic and pharmacological perturbations of MG further established a causal link between the reduced *PICALM* expression, LD accumulation, and phagocytosis deficits. Our work elucidates the selective LOAD vulnerability in microglia for the *PICALM* locus through detrimental LD accumulation, providing a neurobiological basis that can be exploited for developing novel clinical interventions.

Alzheimer’s disease (AD) is the most common cause of dementia and afflicts millions worldwide^8^. Despite decades of studying the pathophysiology of amyloid beta (Aβ) and tau lesions, effective treatment for AD is still lacking. Late-onset AD (LOAD) risk contains a strong genetic component, with ~70% heritability^9^. Recent genome-wide association studies (GWAS) have identified 75 genome-wide significant (*P* < 5×10^−8^) loci for LOAD^1–6^, offering unprecedented opportunities to elucidate the disease biology and facilitate therapeutic strategies for more tailored clinical interventions.

Investigating the functional consequences of LOAD-associated genetic variants in disorder-relevant cell types is essential for understanding the disease mechanisms. Other than the well-established *apolipoprotein E* (*APOE*) locus that contains a protein-coding risk variant (i.e., *APOE e4* allele, *APOE4*) of strong effect size, most LOAD GWAS risk variants are in the noncoding regions of the genome, likely regulating gene expression with smaller effect sizes^9^. Furthermore, recent single-cell sequencing studies of AD postmortem brain tissues implicate disease vulnerability to both neurons and glia at different AD progression states^10–15^. Recent studies of human brain microglia (hMG) have found many LOAD risk variants associated with gene expression (eQTLs)^16–19^. However, such association-based mapping approach does not directly implicate functional or causal disease variants. The functional link between disease risk variants and LOAD-relevant cell phenotypes remains largely unestablished.

Most mechanistic studies of AD have focused on two hallmarks, Aβ and tau lesions. Recently, lipid droplets (LD) – cellular organelles containing lipids such as glycerolipids and cholesterol – have been suggested to play important roles in ageing and neurodegenerative disorders like AD^20–25^. *APOE4*, the strongest genetic risk factor for LOAD, has recently been shown to impair neuron-glia lipid metabolism and cause LD accumulation in glia^23,26^. *APOE4* can also elevate cholesterol synthesis and suppress lysosomal gene activity, leading to lipid accumulation in human glia^27^. Moreover, *APOE4*-associated LD accumulation in oligodendrocytes and MG has been found to modulate neuron function and brain memory^28–30^. However, whether other LOAD GWAS risk loci may similarly confer disease risk by affecting lipids and cholesterol accumulation is unknown.

We have recently developed an allele-specific open chromatin (ASoC) mapping approach that enables a comparison of differential chromatin accessibility of the two alleles of a heterozygous disease risk variant in the same sample, thus directly identifying likely functional disease variants affecting chromatin accessibility and gene expression^7,31^. Here, harnessing the ASoC approach, we systematically identified functional LOAD risk variants in human iPSC-derived MG (iMG), astrocytes (iAst), and other neural cell types. For a strong LOAD risk locus encompassing a gene encoding *phosphatidylinositol binding clathrin assembly protein* (*PICALM*), we found that the LOAD risk variant rs10792832 altered chromatin accessibility to a transcription factor (TF) PU.1 (SPI1) binding site specifically in iMG. *PICALM* has been previously shown to function in autophagy and clathrin-mediated endocytosis^32,33^. *PICALM* can also mediate Aβ endothelial transcytosis and clearance^34^ and rescue endocytosis deficits in *APOE4* astrocytes^35^. However, the MG-specific role of *PICALM* and its link to the LOAD risk allele has not been established. Here, we identified a possible causal link between the LOAD risk allele of rs10792832 and reduced *PICALM* expression, LD accumulation, and phagocytosis deficits in MG.

## Results

### Chromatin accessibility mapping of functional LOAD risk variants in human iMG

**Extended Data Fig. 1.**
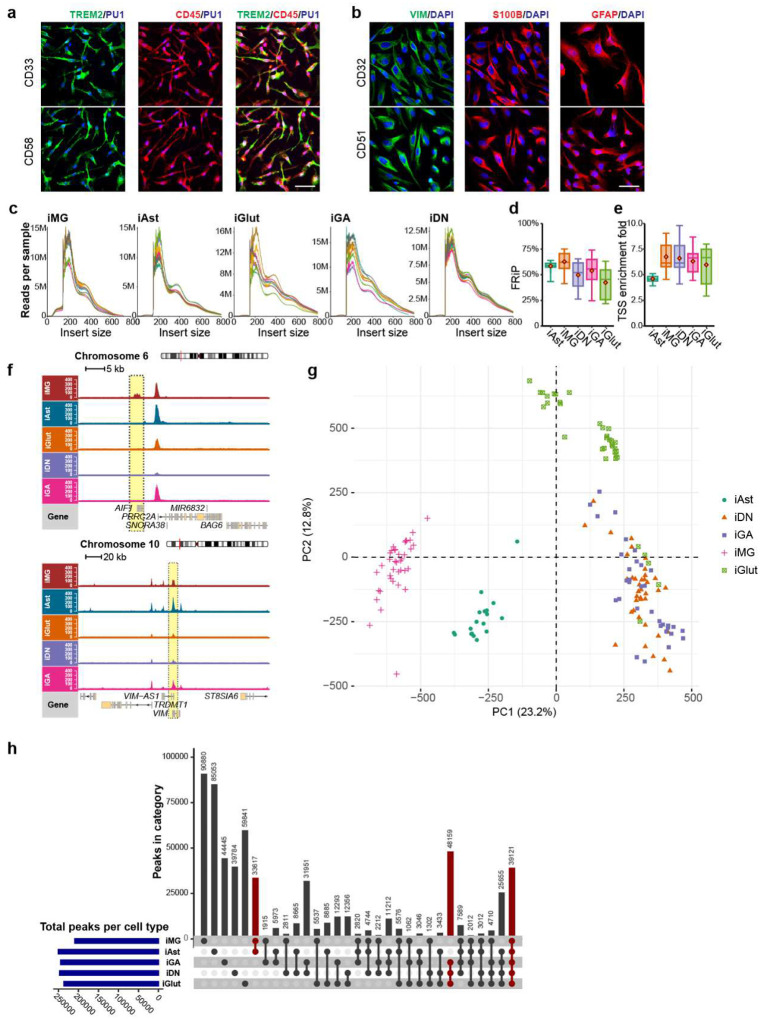
ATAC-seq quality control (QC) and OCR peak calling of each cell type. (a) Representative images of IF staining of iMG (TREM2+CD45+PU1+) used for ATAC-seq. (b) Representative images of IF staining of iAst (S100B+VIM+GFAP+) used for ATAC-seq. (c) ATAC-seq fragment size distribution plots show periodical nucleosome-free region patterns that are similar between samples and different iPSC-derived cell types. (d) and (e) show comparable and acceptable according to the ENCODE ATAC-seq guideline version 4: FRiP (fragment reads in peaks) score > 0.3, TSS (transcription start site) annotation > 5 across cell types. (f) iMG-specific OCR peak for AIF1 (also known as IBA1) and much stronger peaks for VIM in iAs than in other cell types. (g) PCA clustering of human iPSC-derived MG, Ast, NGN2-Glut, GABAergic, and dopaminergic neurons. Normalized ATAC-seq reads derived from a 501-bp union peak set from all five cell types (622,987 peaks in total) were used for PCA. (h) UpSet plot showing the cell type-specific ATAC-seq peaks and shared peaks across different cell types.

**Fig. 1.**
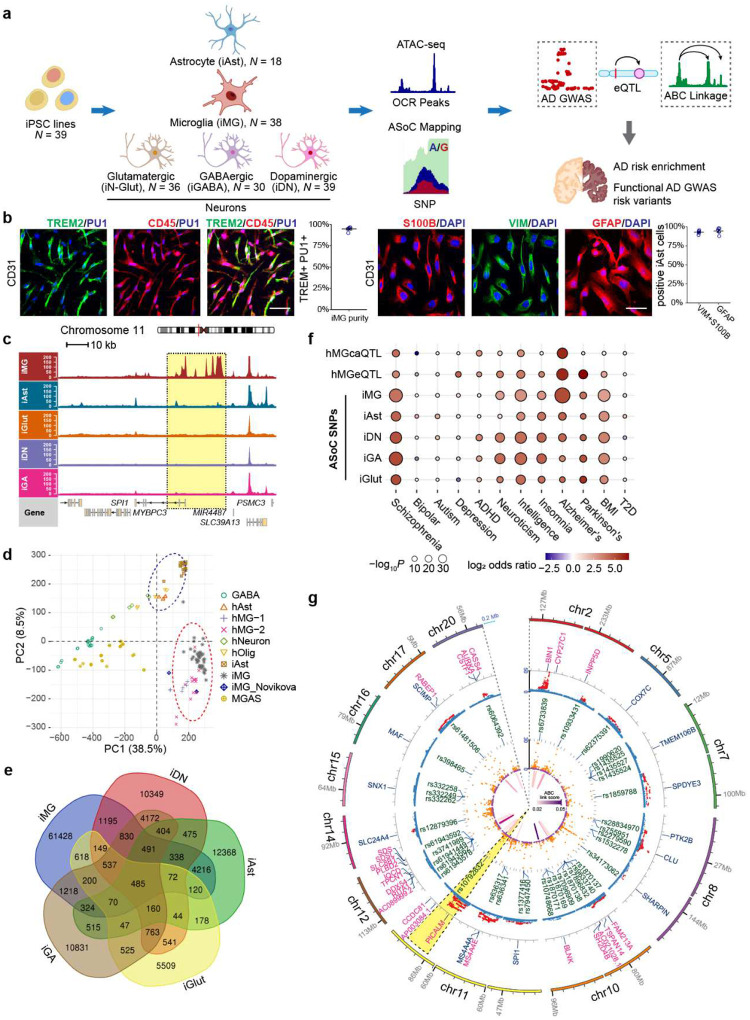
Chromatin accessibility landscape and ASoC mapping in human iMG and other cell types inform functional noncoding GWAS risk variants of LOAD. (a) Schematic of experimental design of ASoC mapping. (b) Immunofluorescence (IF) staining and purity of iMG (TREM2^+^CD45^+^PU1^+^) and iAst (Vimentin^+^/GFAP^+^/S100β^+^). (c) MG-specific ATAC-seq peaks in open chromatin regions of a known MG-specific gene *SPI1*. (d) PCA of ATAC-seq peak accessibility of iMG and iAst samples from the current study in comparison to the reported datasets of human brain MG and Ast (circled areas) or iAst as well as other brain cell types^18,39^. 210,833 peaks from the previous studies^18,39^ were used for PCA. GABA, GABAergic neurons; hOlig, human oligodendrocytes; MGAS, the mixture of microglia and astrocytes. (e) Venn diagram of ASoC SNPs in each cell type. (f) Enrichment of ASoC SNPs (current study) and the reported hMG caQTL or eQTL for GWAS risk of LOAD, NPD, and other complex traits. GWAS datasets are listed in Extended Data Table 8. (g) Circos plot of 19 LOAD risk loci with 38 GWAS risk SNPs that are also ASoC SNPs in iMG. Tracks from inside to outside circles: LOAD risk loci (*n*=9) with ABC enhancer/ASoC SNP links to at least one target genes, FDR (-log_10_) of ASoC SNPs, LOAD risk SNPs that are also ASoC SNPs, LOAD GWAS association *p*-values (-log_10_), index LOAD risk genes, chromosomal regions of LOAD risk loci.

To systematically identify functional LOAD GWAS risk variants, we carried out an assay for transposase-accessible chromatin using sequencing (ATAC-seq) and ascertained allelic imbalance of chromatin accessibility (i.e., ASoC)^7,31^ at heterozygous LOAD risk SNP sites (Fig. 1a). We performed ATAC-seq in human iMG^36^ (*n*=38 donors) and iAst^37^ (*n*=18 donors), two cell types relevant to AD pathogenesis (Fig. 1b, Extended Data Fig. 1a–b). Additionally, we substantially expanded our existing ATAC-seq dataset^7,31^ of iPSC-derived glutamatergic (iGlut, *n*=36), GABAergic (iGABA, n=30), and dopaminergic neurons (iDN, *n*=39) (Fig. 1a, Extended Data Fig. S1c-e, Extended Data Table 1). ATAC-seq peak calling^38^ identified 202,019 to 267,119 open chromatin regions (OCRs) for each cell type.

We confirmed cell type-specific OCRs for marker genes of MGs (e.g., *AIF1*, *SPI1*) and Ast (e.g., *VIM*) (Fig. 1c, Extended Data Fig. 1f). Principal component analysis (PCA) of ATAC-seq results showed clear separation of iMG and iAst from neuronal samples (Fig. 1d, Extended Data Fig. 1g–h). More importantly, PCA showed our iMG and iAst samples were very similar to human brain MG (hMG)^18^, other reported iMG sample^39^ and Ast (hAst) samples^18^ (Fig. 1d), demonstrating epigenomic concordance. Gene ontology (GO) analysis of OCRs further validated the functional relevance of iMG and iAst (Supplementary Fig. 1a–b). Finally, GWAS risk enrichment analysis of OCRs showed that LOAD heritability was strongly enriched in iMG (Enrichment fold = 13.6, *P* = 2.1×10^−15^) and to a lesser extent in iAst (Enrichment fold = 6.3, *P* = 4.9×10^−10^), but not in iPSC-derived different subtypes of human neurons (Supplementary Fig. 1c). In contrast, strong enrichments of neuropsychiatric disorders (NPD) GWAS risk variants were found mainly in neurons (Supplementary Fig. 1c). Thus, our iMG and iAst are epigenetically similar to hMG and hAst with genetic relevance to LOAD.

**Extended Data Fig. 2.**
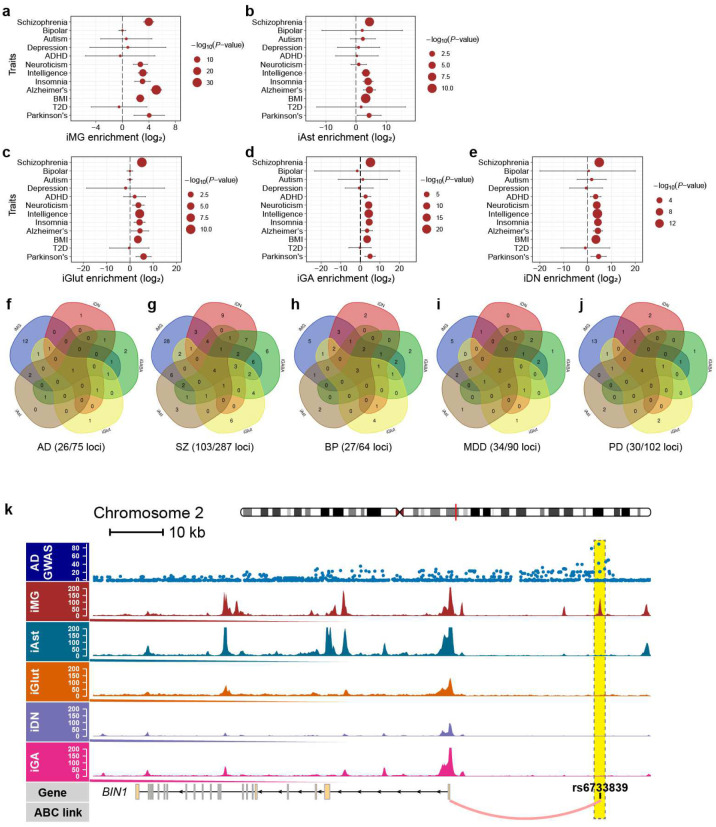
Enrichment of ASoC SNPs for GWAS risk variants LOAD and major NPD in different cell types. (a) to (e) Dot plots showing the fold of GWAS enrichment (log_2_) and *P*-value (-log_10_) of each disorder or trait across different cell types. The analysis was done by using Torus (see Method). (f) to (j) Number of GWAS risk loci with at least one of the GWAS index risk SNPs or their proxies showing ASoC in different cell types for LOAD, SZ, BP (bipolar), MDD (major depressive disorder), and PD. Note the LOAD GWAS risk loci with functional LOAD risk SNPs that show ASoCs are predominately found in iMG (19/26 loci). (k) The GWAS risk SNP rs6733839 at the *BIN1* locus is an ASoC SNP within an iMG-specific OCR peak located in an enhancer assigned to target gene *BIN1* by ABC analysis.

We then identified functional ASoC SNPs in OCRs for each cell type and evaluated their genetic relevance to LOAD. In total, we identified the largest number of ASoC SNPs (*n*=72,291) in iMG and between 15,698 to 31,653 ASoC SNPs in other cell types, of which a large proportion are cell type-specific (Fig. 1e, Extended Data Table 2–6, Supplementary Fig. 2a). Given the similar number of OCR peaks in different cell types, the high number of ASoC SNPs in iMG suggests microglia-specific chromatin accessibility regulation. Similar to the genomic characteristics of neuronal ASoC SNPs^7,31^, 25–30% of ASoC SNPs in iMG and iAst are adjacent to gene promoters (within 5 kb), while most ASoC SNPs are in distal enhancer regions (>50 kb) (Supplementary Fig. 2b–c). GO-term enrichment analysis of ASoC SNPs in iMG and iAst further indicated their functional relevance: ASoC SNPs in iMG tend to be near genes related to immune activation and phagocytosis (Supplementary Fig. 2d–e). Additionally, iMG ASoC SNPs are strongly enriched for hMG caQTLs and eQTLs (Extended Data Tables 2, 7, Supplementary Fig. 2f–h), suggesting they likely alter chromatin accessibility and affect gene expression. Finally, we evaluated the enrichment of ASoC SNPs for GWAS risk variants using TORUS^7^ (Fig. 1f, Extended Data Fig. 2a–e, Extended Data Table 8). We found that neuronal ASoC SNPs were more enriched for GWAS variants of schizophrenia (SZ), neuroticism, and intelligence, consistent with our previous observations^7^. On the other hand, iMG showed the strongest LOAD GWAS risk enrichment (35-fold, *P* = 1.04×10^−33^).

**Fig. 2.**
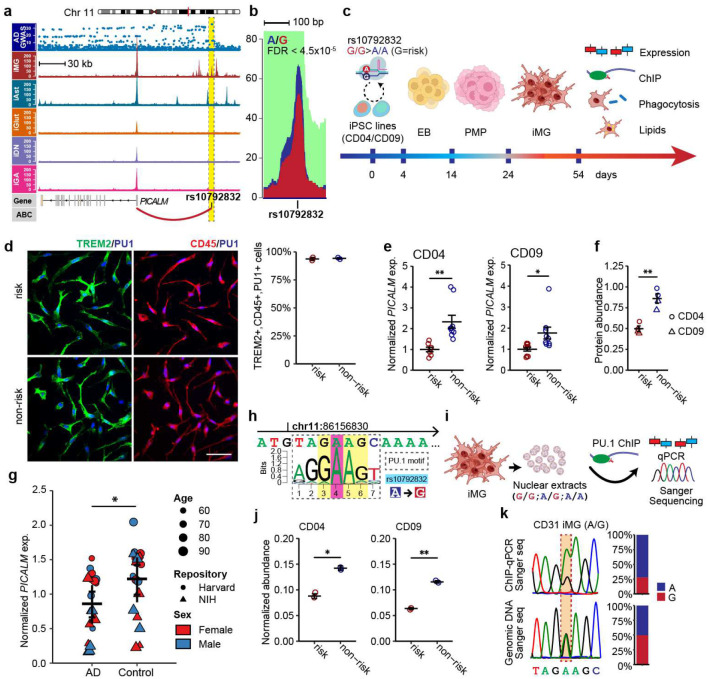
LOAD risk SNP rs10792832 at the *PICALM* locus showed iMG-specific ASoC, and the risk allele is associated with reduced PU.1 binding and *PICALM* expression. (a) LOAD risk SNP rs10792832 shows ASoC and is in an iMG-specific OCR peak. Only iMG samples heterozygous for rs10792832 were used, merged, and down-sampled to 100 M reads for normalization. Note the ASoC SNP showed the strongest GWAS association and is in an upstream enhancer assigned to target gene *PICALM* by ABC analysis. (b) Different ATAC-seq reads of the two alleles of the ASoC SNP rs10792832. ASoC pile-up plot range is 86156600–86157000. A allele, dark blue; G allele, dark red. (c) Schematic design of CRISPR/Cas9 editing of rs10792832 in iPSC (two donor lines CD04 and CD09), iMG differentiation and cellular phenotypic assays. For each line, two isogenic pairs of human iPSC clones were generated and used for RNA-seq and qPCR assay of *PICALM* expression, and all other cellular assays used only one of the two edited clones. (d) IF staining of the CRISPR-engineered isogenic iMG carrying the risk or non-risk allele of rs10792832 (left panels) shows high and similar purity of iMG (TREM2^+^/CD45^+^/PU.1^+^) (right panel). Scale bar: 50 μm. (e) qPCR shows reduced *PICALM* expression in iMG carrying risk alleles of both isogenic pairs (CD04 and CD09). Two biological replicates each with three technical replicates. Expression was normalized to *GAPDH*. Error bars, SEM. (f) Western blot shows risk allele reduced PICALM protein expression. (g) Reduced *PICALM* expression in grey matter of AD patients’ postmortem brains. (h) The LOAD risk allele of rs10792832 is predicted to disrupt PU.1 (SPI1) binding motif (MA0080.2). (i) Schematic design of ChIP-qPCR to assay PU.1 binding. (j) The LOAD risk allele G of rs10792832 shows reduced PU.1 binding in qPCR of ChIP products. Homozygous iMG was used for comparing the allelic effect. (k) Heterozygous iMG (A/G) shows a higher Sanger-sequencing peak of allele A (non-risk) than G (risk). Two-sided unpaired Student’s *t*-test with unequal variance was used in all comparisons.

To assess the degree to which ASoC SNPs can help prioritize GWAS risk variants for LOAD and other NPD, we co-localized ASoC SNPs in each cell type with GWAS index SNPs and their linkage disequilibrium proxies (*r*^2^≥0.8) (Extended Data Tables 2–6). Across all cell types, 29~37% of GWAS risk loci had at least one disease-associated ASoC SNP (Extended Data Fig. 2f–j). For LOAD, out of the 26 risk loci that can be functionally interpreted by ASoC SNPs, 20 loci with 38 ASoC/LOAD risk SNPs were accounted for by iMG (Fig. 1g, Extended Data Fig. 2f, Extended Data Table 2). To further identify the target genes for these ASoC/LOAD risk SNPs in iMG, we integrated our iMG ASoC data with the hMG enhancer-promoter linkages defined by activity-by-contact (ABC) analysis^18^ (Extended Data Table 9). We found that 13 ASoC/LOAD risk SNPs of 9 risk loci can be assigned to a target gene (Fig. 1g), including the two loci with the strongest associations with LOAD: *BIN1* locus for which the functional linkage between rs6733839 and *BIN1* has been well established (Extended Data Fig. 2k)^19,40^, and *PICALM* locus with previously unknown functional links between the rs10792832 and its target gene *PICALM* (Fig. 1g, 2a–b).

### The LOAD risk allele of rs10792832 reduces *PICALM* expression *via* altering PU.1 binding

**Extended Data Fig. 3.**
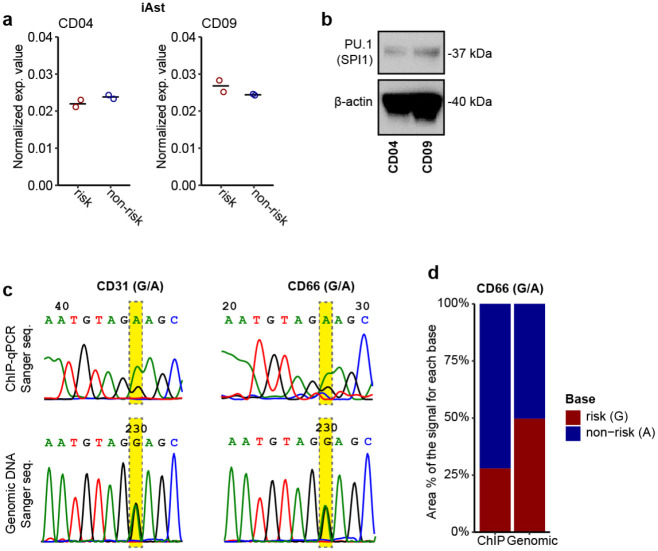
The effect of the LOAD risk allele on *PICALM* expression in iAst and PU.1 binding in iMG. (a) The risk allele of rs10792832 does not affect *PICALM* expression iAst. (b) PU.1 antibody validation in PMP cells from which iMG were further differentiated. (c) The allele A of rs10792832 exhibits higher Sanger sequencing peaks for PU.1 ChIP-seq PCR products of iMG from two iPSC lines heterozygous for rs10792832. Note the equal peak height of the two alleles for genomic PCR products of the same heterozygous samples as in the top panels. (d) Quantifying allelic ratios of Sanger sequencing peaks (c) for genomics DNAs and the ChIP-PCR products of iMG heterozygous for rs10792832.

Among multiple SNPs in complete linkage disequilibrium that similarly show the strongest GWAS association with LOAD (Fig. 2a, top panel), rs10792832 is the only one that showed strong ASoC (Fig.1g, 2b) in an iMG-specific OCR (Fig. 2a). To study the function of the risk allele of rs10792832, we carried out CRISPR/Cas9 editing of human iPSC lines of two non-AD donors (CD04, CD09) by altering risk genotype (G/G) to non-risk genotype (A/A) (Fig. 2c, Supplementary Fig. 3a–e). We then differentiated the two pairs of CRISPR-engineered iPSC lines into iMG (TREM2+/CD45+/PU.1+) (Fig. 2d). We found that the risk allele reduced the expression of *PICALM* by ~40% (Fig. 2e–f) but not in iAst (Extended Data Fig. 3a).

**Extended Data Fig. 4.**
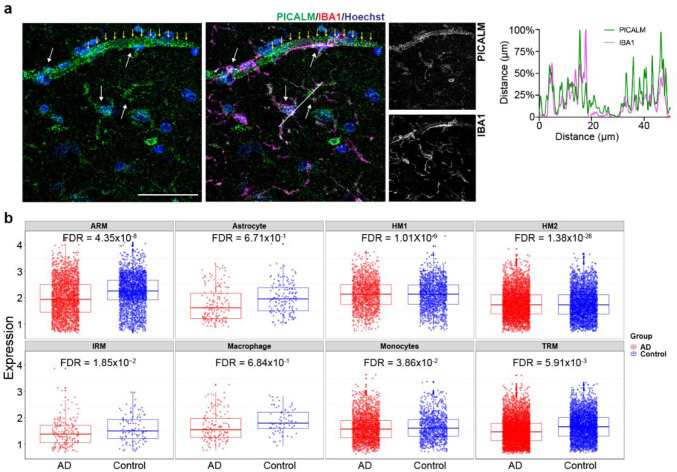
PICALM expression in human postmortem brains and mouse brain cell types. (a) Human post-mortem brain sections from an asymptomatic patient were stained with antibodies against PICALM (green) and IBA1 (magenta). White arrows indicate PICALM expressed in MG, and yellow arrows indicate PICALM expressed in a blood vessel. Line-scan (kymography) analysis (right) demonstrates the overlapping expression. The white line in IBA1 composite image shows the location of the line scan. (b) Single-cell DE analysis of the dorsolateral prefrontal cortex of LOAD and control brains shows reduced PICALM expression in different types of MG but not in Ast, monocytes, or macrophages of LOAD brains^41^. ARM, activate responsive microglia; IRM, interferon response microglia; HM1, homeostatic microglia; TRM, transitioning response microglia. (c) Reduced PICALM expression in cortical MG but not Ast of APPswe/PS1dE9 model compared to non-transgenic control mouse (Non-Tg)^70^.

To assess whether the expression of *PICALM* is altered in human LOAD, we examined the expression of *PICALM* in post-mortem brains of AD patients and controls. We first confirmed the *PICALM* expression in IBA1^+^ MG of the post-mortem human brain (Extended Data Fig. 4a). AD patients showed a reduction of *PICALM* expression in the grey matter (Fig. 2g, Extended Data Table 10). Data mining of single-cell RNA-seq datasets of the human frontal cortex also showed reduced *PICALM* expression in homeostatic or activate-responsive MG but not in Ast^41^ of AD patients (Extended Data Fig. 4b). The reduced expression of *PICALM* in brain MG of AD patients is in accordance with our observed transcriptional effect of the LOAD risk allele of rs10792832 in iMG.

We next examined how the LOAD risk allele of rs10792832 could reduce *PICALM* expression. We found that rs10792832 is within the predicted binding site for a key myeloid-specific transcription factor PU.1, which is encoded by another strong LOAD risk gene *SPI1*^6,9^, with the risk allele G predicted to disrupt the consensus binding motif of PU.1 (Fig. 2h). To empirically test whether the risk allele G of rs10792832 disrupts the PU.1 binding, we performed chromatin immunoprecipitation (ChIP) assay to enrich the PU.1-binding OCRs in iMG of the CRSIPR-edited isogenic iPSC lines, followed by qPCR to assess any differential PU.1 binding between the two alleles (Fig. 2i–j, Extended Data Fig. 3b–d). We found that risk allele G substantially reduced PU.1 binding at the SNP site (Fig. 2j). We also performed ChIP for PU.1 in iMG of two different iPSC lines heterozygous for rs10792832, followed by direct Sanger sequencing to compare the PU.1 binding capacity of the two alleles in the same sample (Fig. 2i, 2k, Extended Data Fig. 3c–d). We found that the risk allele G only retained ~40% of the PU.1 binding capacity of allele A. Together, these data provide compelling evidence that the LOAD risk allele of rs10792832 reduces *PICALM* expression specifically in MG by disrupting the binding of PU.1.

### Reduced *PICALM* expression in iMG impairs phagocytosis of Aβ and myelin debris

**Extended Data Fig. 5.**
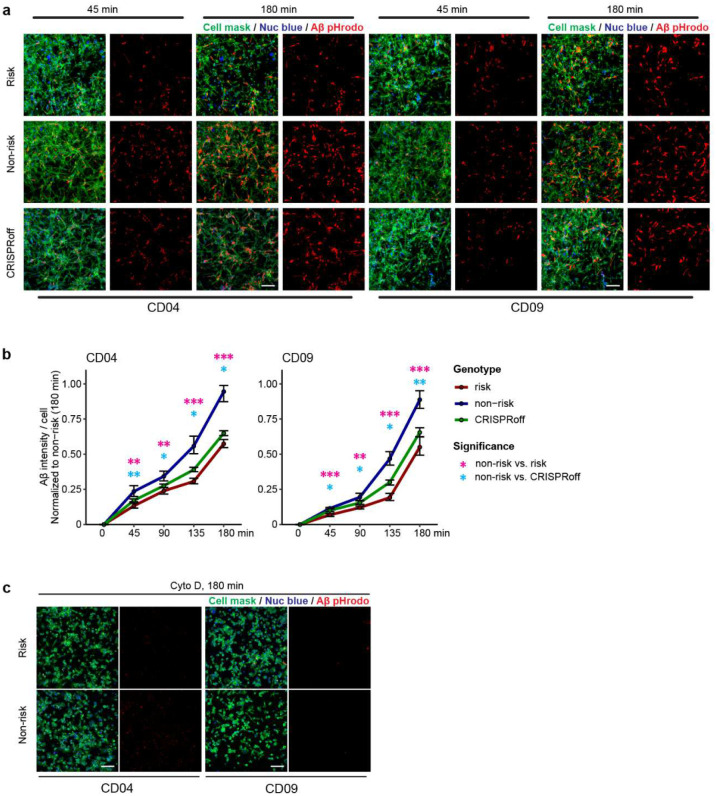
LOAD risk allele of *PICALM* impairs MG phagocytosis of Aβ. (a) Representative fluorescence images of iMG showing time-dependent phagocytosis of Aβ-pHrodo (red). (b) Quantification of Aβ-pHrodo phagocytosis in iMG of CD04 and CD09 lines. Data are from 2–3 independent wells of 4 FOVs each. Two-side unpaired *t*-test was used; * *P*<0.05, ** *P*<0.01, ***, *P*<0.001; error bar, SEM. (c) Cytochalasin D (10 μM) nearly completely inhibits phagocytosis. Cell mask stains for the plasma membrane and nuc blue stains nucleus in living cells. Time shows min after adding myelin pHrodo. Scale bar, 100 μm.

**Fig. 3.**
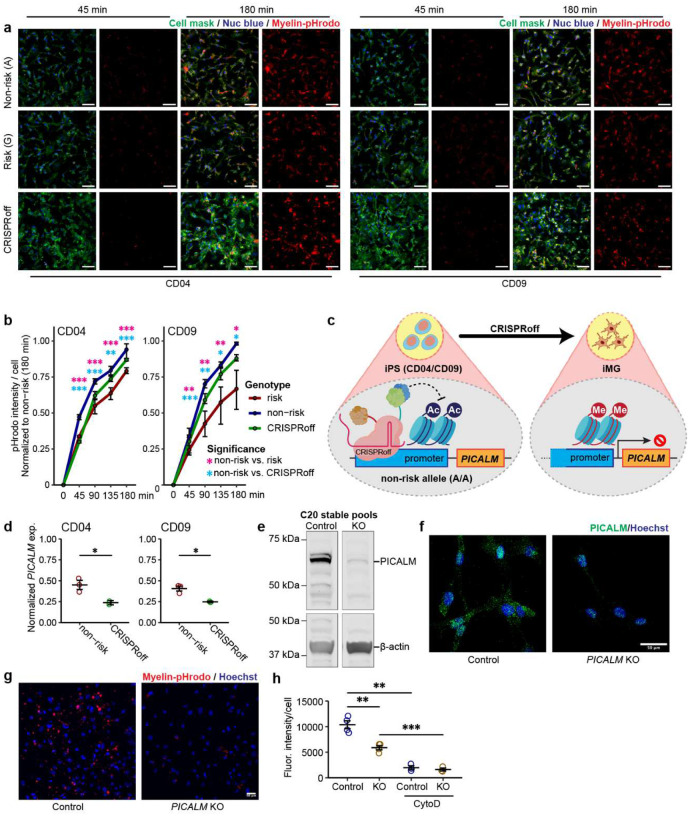
LOAD risk allele of *PICALM* impairs MG phagocytosis of myelin. (a) Representative fluorescence images of iMG showing time-dependent phagocytosis of myelin-pHrodo (red). Cell mask stains for the plasma membrane and nuc blue stains nucleus in living cells. Time shows minutes after adding myelin pHrodo. Scale bar, 100 μm. (b) Quantification of myelin-pHrodo phagocytosis in CD04 and CD09. Data are from 3 independent wells, each with 2–3 fields of view (FOV). (c) Schematics of CRISPRoff to knock down *PICALM* expression for CD04 and CD09 with *PICALM* non-risk alleles. (d) qPCR result shows CRISPRoff-induced reduction of *PICALM* in iMG of both lines. Each dot represents an independent cell culture, each with 3 technical replicates. Expression was normalized to *GAPDH*. Error bars, SEM. (e) and (f) *PICALM* KO by CRISPR/Cas9 editing C20 cells. The gRNA targeted at exon1 depleted PICALM expression in immunoblot (e) and immunofluorescence labelling (f). (g) and (h) *PICALM*-KO C20 cells show decreased phagocytosis of pHrodo-conjugated myelin. Scale bar, 50 μm. In (h), each dot represents a biological replicate. Cytochalasin D (10 μM) was used to show the specificity of phagocytosis. In (b) and (h), Student’s *t*-test (2-tailed, heteroscedastic) was used; * *P*<0.05, ** *P*<0.01, ***, *P*<0.001; error bar, SEM.

The functional relevance of the LOAD risk allele at the *PICALM* locus is unknown. Given the importance of MG phagocytosis in AD pathogenesis^42,43^ and the possible role of PICALM in clathrin-mediated endocytosis in non-MG cells^32,44^, we hypothesized that the LOAD risk allele of rs10792832 affects IMG phagocytosis through the reduced *PICALM* expression. We first examined the phagocytosis of pHrodo-conjugated myelin in CRISPR-engineered iMG carrying the LOAD risk allele or non-risk allele. For both iPSC lines, myelin-pHrodo fluorescence intensity was significantly reduced by ~20% in iMG carrying the risk allele compared to the non-risk allele (Fig. 3a–b). We also tested the phagocytic activity of iMG using pHrodo-labelled Aβ aggregates and found similarly that harbouring the LOAD risk allele displayed a significant reduction of pHrodo fluorescence intensity by up to 40% (Extended Data Fig. 5a–b).

To assess whether the reduced phagocytic activity of iMG carrying the LOAD risk allele was due to the reduced *PICALM* expression, we used CRISPRoff to engineer iPSC lines carrying the non-risk allele to repress *PICALM* expression in iMG (Fig. 3c). CRISPRoff achieved a magnitude of *PICALM* expression reduction that mimics the regulatory effect of the risk allele rs10792832 for both iPSC lines (Fig. 2e, 3d). We found that iMG with CRISPRoff exhibited a reduced capacity for phagocytosis of myelin-pHrodo and Aβ-pHrodo compared to iMG carrying the non-risk allele (Extended Data Fig. 5a–b), suggesting a direct link between reduction of *PICALM* and iMG phagocytosis.

To further corroborate the effect of *PICALM* expression levels on phagocytosis in MG, we performed an independent CRISPR/Cas9 editing of an immortalized hMG cell line (C20)^45^ to generate stable pools of *PICALM* knockout (KO) cells (Fig. 3e–f). The loss of *PICALM* expression in C20 cells did not perturb the Golgi apparatus (Giantin), the distribution of early endosomes (EEA1), AP-4 containing secretory and endocytic vesicles, or internalization of transferrin and cholera toxin by endocytosis (Supplementary Fig. 4). However, *PICALM*-KO cells showed significantly reduced phagocytosis of myelin-pHrodo (50% reduction) compared to wild type (WT) (Fig. 3g–h), which was consistent with our observed phagocytosis deficit in iMG with reduced *PICALM* expression (Fig. 3a–b). These results suggest impaired MG phagocytosis of damaged myelin and Aβ aggregates may be a causal mechanism of the LOAD risk allele at the *PICALM* locus.

### Transcriptomic profiling of phagocytosis-deficit iMG reveals lipid dysregulation

**Fig. 4.**
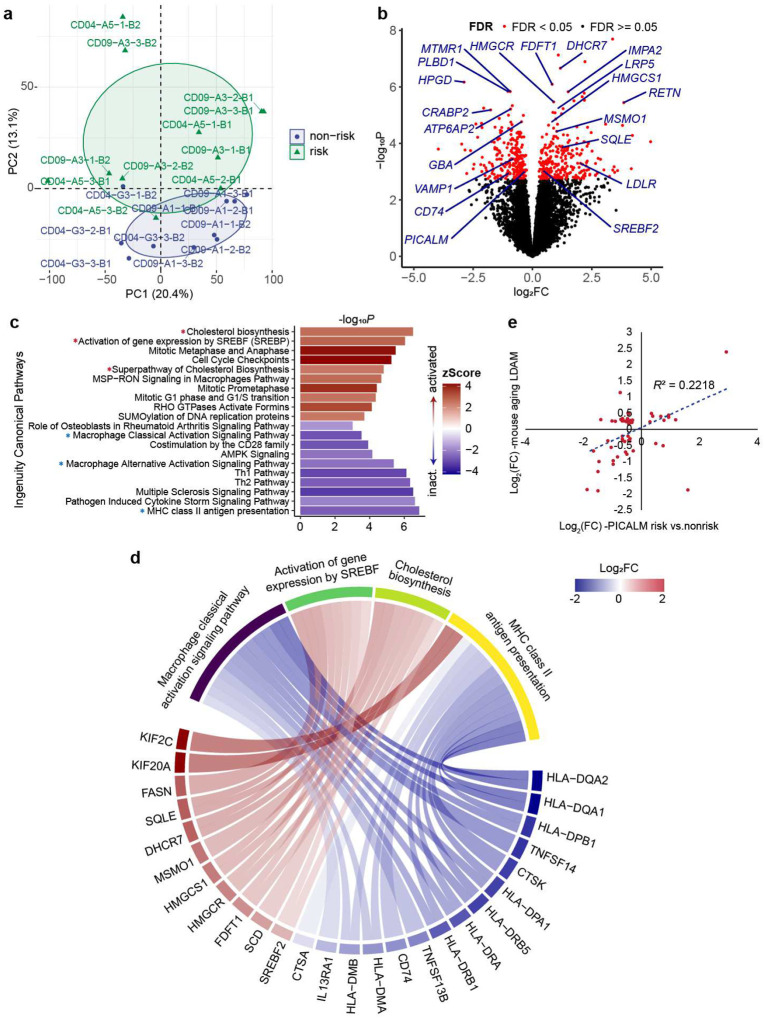
Transcriptomic effects of the *PICALM* LOAD risk allele in iMG. (a) PCA of RNA-seq samples of iMG derived from the isogenic pairs of CRISPR-engineered iPSC lines carrying the *PICALM* risk or non-risk alleles. Samples are from 2–3 independent cultures of each isogenic pair from 2 different batches for CD04 and CD09 lines. Expression of 13,947 genes was used for PCA. (b) Volcano plot shows DE genes in iMG carrying the LOAD risk allele. (c) Enriched Ingenuity canonical pathways for all DE genes (FDR<0.05). Significantly (FDR<0.05) enriched pathways are ranked by their activated or inactivated Z-scores. (d) Circos plot of DE genes and their expression fold-changes (FC, log_2_ scale) for major gene pathways highlighted in (c). (e) Significant correlation of the expression changes (-log_2_FC) in iMG carrying *PICALM* risk allele (vs. non-risk allele) and in previously reported LD accumulated microglia (LDAM) of the ageing mouse^20^. Plotted are 56 genes showing DE (FDR<0.1) in both RNA-seq datasets.

To study the molecular mechanism underlying the observed phagocytosis deficit in iMG carrying the *PICALM* risk allele, we performed differential expression (DE) analysis of iMG carrying the LOAD risk vs. non-risk allele for both isogenic iPSC pairs (Fig. 4a). We identified 257 upregulated and 244 downregulated genes (FDR < 0.05), including the expected decrease of *PICALM* (Fig. 2e–f), in iMG harbouring the LOAD risk allele (Fig. 4b, Extended Data Table 11). GO-term enrichment analysis of the DE genes showed that the downregulated genes were enriched for GO terms related to MHC class II protein complex and antigen presentation as well as clathrin-coated endocytic vesicle membrane (Extended Data Table 12, Supplementary Fig. 10a), which are important for MG phagocytosis^46^. Surprisingly, more than half of the 20 top-ranked DE genes (including *DHCR7*, *HPGD*, *FDFT1*, *HMGCR*, *PLBD1*, *IMPA2*, *MTMR1*, *RETN*, *ATP6AP2*, *LRP5*, *CRABP2*) were related to lipid metabolic processes (Fig. 4b, Extended Data Table 11). Notably, among these genes, *ATP6AP2* (log_2_FC = −0.92, FDR = 0.004) encodes a transmembrane protein involved in autophagy and LD formation^47,48^. Moreover, among the top 10 enriched GO-terms for upregulated genes, 4 were related to lipid/cholesterol biosynthesis or metabolism (Extended Data Table 12, Supplementary Fig. 5a).

The results from the GO-term enrichment were further confirmed by an independent Ingenuity Pathway Analysis (IPA)^49^. Among the most enriched canonical pathways (Fig. 4c–d), the activated ones are related to lipid metabolism, such as cholesterol biosynthesis, activation of gene expression by SREBF (also known as SREBP, sterol regulatory element binding protein)^50^. In contrast, the inactivated ones included MHC class II antigen presentation and microphage classical/alternative signalling pathways that are relevant to phagocytosis^46,51^. All genes in the activated cholesterol biosynthesis pathway were upregulated (Fig. 4d, Supplementary Data Fig. 5b) in iMG carrying the *PICALM* risk allele. Consistent with the activation of cholesterol biosynthesis^52^, we observed that *SREBF2* but not *SREBF1* was upregulated in iMG with *PICALM* risk allele (Fig. 4b,d). Moreover, activation of SREBP has also been demonstrated to be a downstream effect of cellular reactive oxygen species (ROS)^53^.

We next compared the transcriptomic similarity of iMG carrying the LOAD risk allele to the recently reported LD-accumulated microglia (LDAM) from ageing mice^20^. For the overlapping DE genes (*n*=56) between the two datasets, the expression changes in iMG with the LOAD risk allele and the ageing LDAM were significantly correlated (*r*^2^=0.22) (Fig. 4e, Extended Data Table 14). Enriching activated lipid/cholesterol metabolism pathways in iMG carrying the LOAD risk allele at the *PICALM* locus and its transcriptomic similarity to mouse LDAM suggests a possible functional link between *PICALM* dysregulation and LD formation in MG.

### LOAD risk allele at *PICALM* locus increases LD accumulation in iMG

**Fig. 5.**
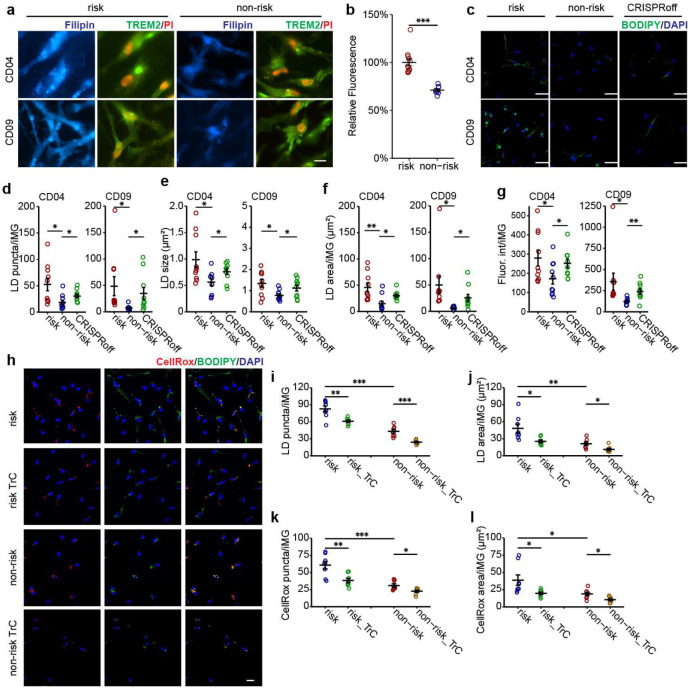
iMG carrying the LOAD risk allele of rs10792832 shows LD accumulation and produces more ROS. (a) IF staining of filipin in day-25 iMG (TREM2^+^) carrying the LOAD risk or non-risk allele of rs10792832. (b) Quantification of filipin fluorescence intensity in (a). PI, propidium iodide for nucleus staining. Scale bar: 50 μm. CD04 and CD09 lines, two replicate wells, each with 2–3 FOV. Student’s *t*-test (2-tailed, heteroscedastic) was used; * *P*<0.05, ** *P*<0.01, ***, *P*<0.001; error bar, SEM. (c) IF staining of LD (BODIPY^+^) in iMG carrying risk or non-risk allele and in CRISPR-off iMG. Scale bar: 50 μm. (d) to (g) Increased LD (BODIPY^+^) area, density per cell, size and fluorescence intensity in iMG carrying the LOAD risk allele (vs. non-risk). Note iMG with *PICALM* CRISPRoff also show increased LD accumulation (vs. non-risk allele), mimicking the effect of the *PICALM* risk allele. CD04 and CD09 lines, 3 replicate wells each with 3–4 FOV. Student’s *t*-test (2-tailed, heteroscedastic); * *P*<0.05, ** *P*<0.01, ***, *P*<0.001; error bar, SEM. (h) fluorescence staining of LD (BODIPY^+^) and ROS (CellRox^+^) with or without pretreatment of Triacsin C (TrC) in iMG carrying risk or non-risk allele. Scale bar: 50 μm. (i) to (l) Increased LD and CellRox density and area in iMG carrying LOAD risk allele (vs. non-risk). TrC reduces both LD formation and CellRox. CD04 line, 2 replicate wells each with 4 FOV. Student’s *t*-test (2-tailed, heteroscedastic); * *P*<0.05, ** *P*<0.01, ***, *P*<0.001; error bar, SEM.

LD accumulation in glial cells is emerging as a possible mediating mechanism for aging and neurodegeneration^20,27,53^. Given the enriched pathways related to lipid metabolism in our transcriptomic analyses, we hypothesized that the LOAD risk allele at the *PICALM* locus might lead to cholesterol accumulation and excess LD formation in iMG. We used filipin to stain intracellular free cholesterol in iMG differentiated from the isogenic CRISPR-edited pairs of iPSC lines carrying the LOAD risk allele (Fig. 5a–b). We found that the fluorescence intensity of filipin staining in iMG carrying the LOAD risk allele was significantly higher than in iMG with the non-risk allele (Student’s t-test, *P* < 1×10^−6^).

Cholesterol esters, derived from cellular cholesterol, are stored in LD along with triacylglycerols^54^. Although filipin only stains free (*i.e*., unesterified) cholesterol, cellular cholesterol accumulation is associated with excess LD^20,27,54^. Thus, we stained iMG using BODIPY, a dye commonly used to detect lipids in LD. For both iPSC lines, we observed a 2 to 7-fold increase of LD in iMG carrying *PICALM* risk allele, as measured by LD area, density, and fluorescence intensity, and to a lesser extent, an increase in LD size (~1.6-fold) (Fig. 5c–g). To confirm the specificity of the BODIPY-labelling of LD, we treated iMG cultures with Triacsin C (TrC) for 18 hours, an inhibitor of long-chain acyl-CoA synthetase that inhibits glycerolipid synthesis and thus LD formation^20,55,56^. TrC effectively reduced BODIPY-labelled LD by 30–50% in iMG with either LOAD risk or non-risk allele (Fig. 5h–j).

To further examine whether the LD accumulation in iMG with the *PICALM* risk allele was due to the reduced expression of *PICALM*, we compared the LD staining in iMG of the LOAD non-risk allele and their derivative iMG with *PICALM* knockdown (KD) by CRISPRoff (Fig. 3c–d). We found that iMG from the *PICALM*-KD line showed significantly more LD accumulation than iMG of the non-risk allele, mimicking the effect of the LOAD risk allele of rs10792832 in iMG (Fig. 5c–g). Independent LD staining by LipidTOX produced similar results to BODIPY-labelling of LD in iMG of the LOAD risk allele, non-risk allele, or non-risk *PICALM* CRISPRoff (Extended Data Fig. 7a–e), further supporting the effect of reduced *PICALM* expression on LD accumulation in iMG. Finally, our observed link between the reduced *PICALM* expression and LD accumulation in iMG was corroborated by detecting LD accumulation in *PICALM*-KO C20 cells (Extended Data Fig. 7f).

LD accumulation in iMG may be due to *de novo* cholesterol synthesis (Fig. 5) and/or excess lipid uptake. To test whether the lipid accumulation in iMG with the LOAD risk allele can also be attributed to lipid uptake, we carried out a lipid transfer assay^57^ in which we first pre-labelled iGlut neurons with Red-C12 overnight and then co-cultured the Red-C12-labelled neurons with iPSC-derived iMG (Extended Data Fig. 8a). We found that the Red-C12 density and area were similar between iMG with the LOAD risk and non-risk alleles (Extended Data Fig. 8b–d) indicating that the LOAD risk allele of *PICALM* does not affect lipids uptake by iMG. The unchanged lipid uptake in iMG with the LOAD risk allele was supported by our RNA-seq data in which *TREM2*, a major lipid receptor specifically expressed by MG, was not differentially expressed between iMG carrying the LOAD risk vs. non-risk alleles (Extended Data Table 11, Supplementary Fig. 5b). To confirm further our observed no-effect of *PICALM* on lipid uptake in iMG, we also examined whether *PICALM* affects neuron-astrocyte lipid transfer and LD formation in human iAst using the same lipid transfer assay system (Extended Data Fig. 8a). Because the LOAD risk allele of rs10792832 does not affect *PICALM* expression in iAst (Supplementary Fig. 3d), we cultured the Red-C12-labeled neurons with iAst carrying the LOAD non-risk allele or *PICALM* non-risk CRISPRoff (Extended Data Fig. 8a). Consistent with the finding in *Drosophila* where *PICALM* facilitates neuron-astrocyte lipid transfer and glial LD formation^24^, iAst with *PICALM* CRISPRoff showed ~50% reduction of BODIPY-labelled LD as well as Red-C12-labeled lipids (Extended Data Fig. 8e–i). These results highlight cell type-specific effects of *PICALM* on lipid transfer and LD formation in iMG and iAst. These results corroborated that reduced expression of *PICALM* by the LOAD risk allele causes MG-specific LD accumulation.

### iMG with LOAD risk allele show elevated reactive oxygen species (ROS)

In ageing mice, LDAM have elevated levels of ROS that may be associated with cellular oxidative stress and age-related neurodegeneration^20^. In *Drosophila*, neuronal ROS is required in conjunction with LD accumulation to promote neurodegeneration^53^. Our RNA-seq data of iMG carrying the *PICALM* LOAD risk allele also revealed the activation of the SERBP pathway, an indicator of elevated ROS level^53^. We thus hypothesized that the LOAD risk allele of *PICALM* increases ROS in iMG. To test the hypothesis, we treated iMG cultures with CellROX, a non-fluorescent dye that exhibits deep-red fluorescence upon oxidation by ROS and also stained the cells with BODIPY to visualize LD (Fig. 5h). We found that iMG carrying the LOAD risk allele showed more than 2-fold of CellROX staining (Fig. 5i–l), with CellROX and LD staining largely colocalized (Fig. 5h). To examine whether LD accumulation releases ROS levels in iMG or vice versa, we treated iMG with TrC to block LD formation. We found that the TrC-treated iMG exhibited a substantial reduction of CellROX (Fig. 5i–l), implying a direct link between LD accumulation and the increase of ROS in iMG.

Lipids are often peroxidated in the presence of ROS and mediate cellular oxidative stress^53^. We thus examined whether the accumulated lipids in iMG were peroxidated. We stained iMG with BODIPY-C11(581⁄591) (Extended Data Fig. 9a), a fluorescent lipid peroxidation sensor that shifts its fluorescence emission from red to green when peroxidated lipids are present. We observed that lipids in a large proportion of LD in iMG were peroxidated (Extended Data Fig. 9b). Consistent with the result from lipid staining with regular BODIPY (Fig. 5c–g), iMG with the LOAD risk allele exhibited about 3-fold more peroxidated lipids accumulation compared to iMG with the non-risk allele, and the LD accumulation was reduced by TrC treatment (Extended Data Fig. 9b–d). The increased ROS and the accumulation of peroxidated lipids suggest a state of cellular stress of iMG carrying the LOAD risk allele of *PICALM*.

### LD accumulation in iMG carrying the LOAD risk allele impairs phagocytosis

**Fig. 6.**
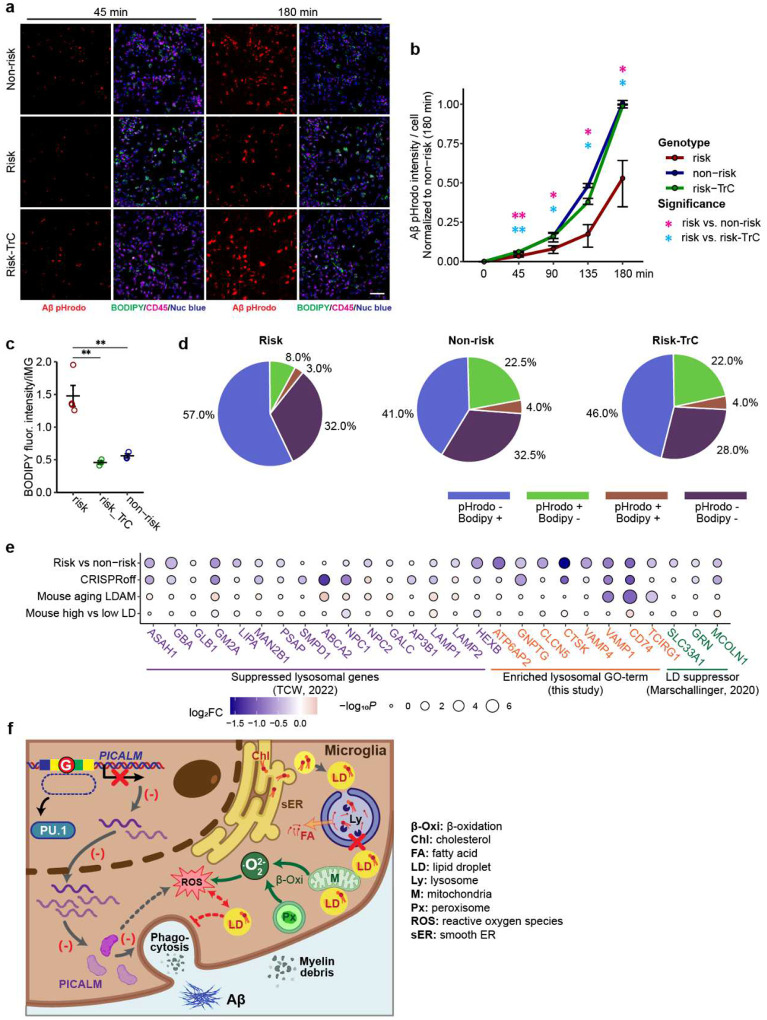
LD accumulation impairs iMG phagocytosis and possible mechanism. (a) Representative fluorescence images show time-dependent phagocytosis of Aβ-pHrodo and LD accumulation (BODIPY^+^) in iMG (CD45^+^) carrying the LOAD risk allele or non-risk allele of *PICALM* in the presence or absence of TrC. Scale bar: 100 μm. (b) Reduced Aβ-pHrodo intensity in iMG carrying the LOAD risk allele (vs. non-risk) can be rescued by TrC treatment. (c) TrC treatment rescues the LD accumulation in iMG carrying the LOAD risk allele to a level similar to that in MG carrying the non-risk allele. (d) Pie charts show the proportion of iMG (CD45^+^) stained positive for Aβ-pHrodo, BODIPY, or both from co-localization analysis of the fluorescence images in (a). Note the Aβ-pHrodo^+^/BODIPY^+^ iMG are rare in each type of iMG, and TrC treatment rescues the phagocytosis deficit in iMG carrying the LOAD risk allele by mainly converting BODIPY^+^ iMG to phagocytic cells without LD (Aβ-pHrodo^+^/BODIPY^−^). CD09 line was used, 3 replicate wells each with 1–2 FOV. Student’s *t*-test (2-tailed, heteroscedastic); * *P*<0.05, ** *P*<0.01, ***, *P*<0.001; error bar, SEM. (e) Dysfunctional lysosome may contribute to LD accumulation in iMG carrying the LOAD risk allele of *PICALM*. Shown are log_2_FC of known lysosomal genes and LD suppressor genes in iMG carrying the LOAD risk allele or with *PICALM* CRISPRoff (vs. non-risk). (f) Mechanistic insight on the link between risk allele of the LOAD GWAS SNP and the reduced *PICALM* expression, lysosomal dysfunction, LD accumulation, LD peroxidation, cellular ROX level, and phagocytosis deficits in iMG. (−), inhibition; FA: fatty acids; Px: peroxisome; M: mitochondria; sER: smooth ER; Ly: lysosome; Chl: cholesterol.

LDAM in mice show defective phagocytosis^20^; however, whether LD accumulation impairs phagocytosis remains unclear. Our iMG carrying the LOAD risk allele that showed both phagocytosis deficits and LD accumulation provides a tractable cellular model for testing the causal link between the two. We thus carried out a phagocytosis assay of the iMG carrying the LOAD risk allele in the presence or absence of LD inhibitor, TrC, to determine whether the LD accumulation leads to phagocytosis deficits (Fig. 6a–d, Extended Data Fig. 10a–d). We first confirmed the reduced phagocytosis of Aβ (Fig. 6a–b) and myelin (Extended Data Fig. 10a–b) in iMG with the LOAD risk allele. We found that TrC treatment of iMG carrying the LOAD risk allele recovered their capacity of phagocytosis of Aβ or myelin to a level of iMG with the non-risk allele (Fig. 6a–b, Extended Data Fig. 10a–b), which was accompanied by a reduction of LD abundance in iMG carrying the risk allele (Fig. 6a, c and Extended Data Fig. 10a, c), suggesting LD accumulation likely causes the phagocytotic deficit in iMG.

To further corroborate the observed effects of LD accumulation on iMG phagocytosis, we analyzed the colocalization of BODIPY staining (LD accumulation) and pHrodo fluorescence (phagocytosis) (Fig. 6a, d, Extended Data Fig. 10a, d). We found that for iMG with the non-risk allele, BODIPY-negative cells accounted for 85% of the phagocytotic (Aβ/pHrodo+) cells. The LOAD risk allele reduced the proportion of phagocytotic (Aβ/pHrodo+) cells from 26.5% to 11%, while BODIPY- cells increased proportionally (Fig. 6d). Moreover, TrC treatment of iMG carrying the LOAD risk allele shifted the proportions of cells with different BODIPY/pHrodo combinations to a similar pattern of iMG with the non-risk allele (Fig. 6d). Colocalization analysis of phagocytosed myelin and BODIPY in iMG gave similar results (Extended Data Fig. 10d). Collectively, these results support that LD accumulation in iMG harbouring *PICALM* risk allele impairs phagocytosis.

### Lysosomal dysfunction may contribute to LD accumulation in iMG with the LOAD risk allele of *PICALM*

**Extended Data Fig. 6.**
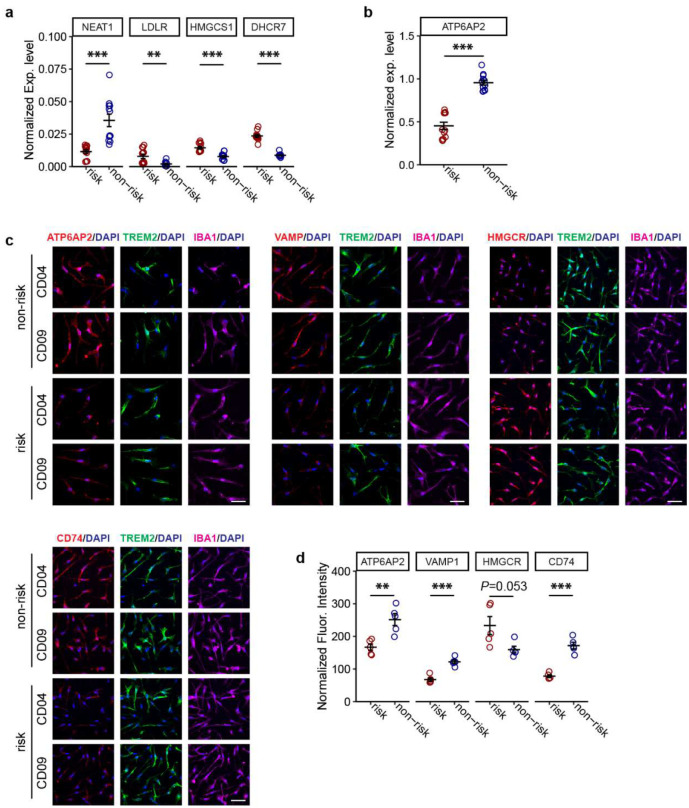
Validation of the selected DE genes by qPCR and IF staining. (a) and (b) qPCR results show reduced expression of *NEAT1* and *ATP6AP2* while increased expression of *LDLR*, *HMGCS1*, and *DHCR7* in iMG carrying the *PICALM* risk allele (vs. non-risk). Two biological replicates, each with three technical replicates. Expression was normalized to GAPDH. Error bars, SEM. (c) IF staining and (d) quantification of mean fluorescence intensity of selected DE genes in day-25 iMG carrying non-risk or risk alleles. The protein expression of lysosomal genes ATP6AP2, VAMP1, and CD74 is reduced while the cholesterol synthesis gene HMGCR expression is increased in iMG carrying the LOAD risk allele. Scale bar, 50 μm. Data are from 2–3 independent wells of 2–3 FOVs each. Student’s *t*-test (2-tailed, heteroscedastic) was used; * *P*<0.05, ** *P*<0.01, ***, *P*<0.001; error bar, SEM.

In ageing mice, it has been proposed that dysfunctional lysosomes may contribute to LD accumulation^20^. For *APOE4*, downregulating the lysosomal gene network is also suggested to contribute to glial lipid/cholesterol accumulation^27^. We thus examined whether LD accumulation in iMG with *PICALM* risk allele may be due to compromised lysosomal function. We found that for those lysosomal network genes downregulated in *APOE4* glia^27^ (Fig. 6e, Extended Data Table 15), 13/15 of them showed reduced expression in iMG carrying the LOAD risk allele (9 with *P* <0.05). iMG with *PICALM* CRISPRoff also showed largely consistent expression changes with those harbouring the LOAD risk allele (Fig. 6e). We also found that the downregulated genes in iMG carrying the *PICALM* risk allele were enriched for the GO-term lysosome (cellular component) (Supplementary Fig. 5a) that shared some downregulated lysosomal membrane genes (*VAMP1*, *CD74*, *TCRIG1*) in mouse ageing LDAM (Fig. 6e, Extended Data Fig. 10e). It is noteworthy among the enriched lysosomal GO-term genes, *ATP6AP2*, one of the most downregulated genes in iMG carrying PICALM risk allele (Fig. 4b, Extended Data Table 11), encodes a transmembrane protein essential for lysosomal degradative functions^48,58^, and mice with mutation of *Atp6ap2* show increased triglycerides and LD in fat body cells^48^. The reduced protein expression of ATP6AP2, VAMP1, and CD74 in iMG carrying the LOAD risk allele of *PICALM* was further independently confirmed by IF staining (Extended Data Fig. 6c–d).

Moreover, three known LD suppressor genes (*SLC33A1*, *MCOLN1*, *GRN*)^20^ all showed reduced expression in iMG carrying the *PICALM* LOAD risk allele (Fig. 6e), of which *MCOLN1* and *GRN* also showed reduced expression in iMG with *PICALM* CRISPRoff (Fig. 6e) and had a role in lysosomal function: MCOLN1 is a ROS sensor in lysosomes that regulates autophagy^59^, and *GRN* encodes the lysosomal protein progranulin that has been implicated in some neurodegenerative diseases^60^. Together, these data support the pivotal role of dysfunctional lysosomes in LD accumulation in iMG carrying the LOAD risk allele of *PICALM* (Fig. 6f).

## Discussion

Our integrative analyses of chromatin accessibility in iPSC-derived major brain cell types with brain QTLs, chromatin interactions (ABC), and LOAD GWAS data identified functional LOAD risk variants accounted for about 1/3 of the known LOAD GWAS risk loci, providing a rich resource for prioritizing functional LOAD GWAS risk variants/genes for biological follow-up. Importantly, we mechanistically tie the putative LOAD causal SNP at the *PICALM* locus to its iMG-specific functional effect on *PICALM* expression, resulting in LD accumulation in iMG (Fig. 6f). Pharmacological perturbation further established a causal link between LD accumulation and the impaired phagocytosis of Aβ and myelin debris in iMG (Fig. 6f). Our work thus elucidates a previously unappreciated MG-specific role for *PICALM* in regulating lipid/cholesterol metabolism that is potentially linked to LOAD pathophysiology.

PICALM has been suggested to function in mouse endothelial cells to clear Aβ^34^. In *Drosophila* glia and rat astrocytes, PICALM is required to store peroxidated lipids sourced from neurons within LD^24^. Our study indicates that PICALM is central in regulating lipid/cholesterol homeostasis and phagocytosis in MG, independent of interactions with neurons. Our transcriptomic analysis of iMG carrying the *PICALM* LOAD risk variant suggests that *de novo* cholesterol and fatty acid (FA) synthesis may contribute to LD accumulation. In support of this, genes in the cholesterol biosynthesis pathway were all upregulated in iMG carrying the LOAD risk allele (Fig. 4b, d). This is consistent with a previous finding in HEK293 cells where the loss of *PICALM* alters the net scavenging of cholesterol^61^. Another major upregulated gene, *SREBF2*, controls the expression of genes involved in cholesterol^50^ and FA^62,63^ synthesis. In addition, the transcriptomic profiling also suggests a lysosomal dysfunction that impairs lipid processing (Fig. 6e–f). Importantly, the expression of several known lysosomal genes (*MCOLN1*, *GRN*, *VAMP1*, *CD74*, *TCRIG1*) whose reduced expression is associated with LD accumulation in mouse MG^20^ was also reduced in our iMG carrying the LOAD risk allele (Fig. 6e). Given that the elevated cholesterol and FA synthesis and the downregulated lysosomal pathway genes have also been observed for the APOE4 allele in astrocyte^27^, LD accumulation due to an upregulation of cholesterol and FA synthesis and/or lysosomal dysfunction in glial cells may be a shared pathophysiological mechanism for major LOAD risk factors.

Harnessing the functional LOAD risk variants identified by our ASoC mapping, we were able to directly tie the *PICALM* risk locus to LD accumulation, impaired phagocytosis, and elevated ROS in iMG. While phagocytosis deficits and ROS elevations have also been observed in mouse LDAM^20^, the causal relationship between LD accumulation, phagocytosis, and ROS is unclear. Here, blocking LD formation with TrC in iMG carrying the *PICALM* risk allele nearly completely rescued phagocytosis deficits (Fig. 6a–d, Extended Data Fig. 10a–d) and significantly reduced the level of ROS (Fig. 5h), suggesting a role of LD accumulation in mediating the observed phagocytosis deficits and ROS elevation (Fig. 6f). However, we also found that LD in iMG contained substantial amounts of peroxidated lipids (Extended Data Fig. 9b–d), which suggests that some ROS may occur first, and the peroxidized lipids are then sequestered in LD^26,53,64,65^. In glia, the main sources of ROS may include peroxisomes and mitochondria that perform β-oxidation of lipids^64–66^. Endoplasmic reticulum (ER) stress can also produce ROS^67,68^. As for how *PICALM* may affect ROS in iMG carrying the *PICALM* risk allele (Fig. 5h), we noted that *PICALM* and some other clathrin adaptor protein complex 2 (AP2) genes had been reported to be protective against ROS production partially through iron regulation^69^.

Hence, a plausible model (Fig. 6f) linking the *PICALM* risk allele to LD accumulation, ROS elevation, and phagocytosis deficits may be: (1) a partial loss of *PICALM* induces ROS and lysosomal dysfunction which leads to elevated lipid/cholesterol synthesis through SREBP pathway, followed by lipid peroxidation and LD formation that is initially protective; (2) over time, the LD formation in the ER and LD accumulation causes an ER stress and overwhelm the mitochondria processing of the lipids, which further elevates ROS; (3) an impaired phagocytosis may be caused by lower PICALM levels, lysosomal dysfunction, and/or impaired energy metabolism. Alternatively, *PICALM* risk allele-induced phagocytosis/endocytosis deficits at an early stage may transiently reduce cellular lipids/cholesterol levels, which in turn activates *de novo* cholesterol synthesis, a process that is accompanied by ROS elevation and lipid peroxidation, leading to LD accumulation in iMG. Future studies to mechanistically link these forward-feedback processes will help understand how the *PICALM* risk allele leads to compromised MG. Nonetheless, given that the *APOE4* allele was recently found to induce LD accumulation, thereby impairing neuronal function^29,30^, our demonstration of the role of the LOAD risk allele at the *PICALM* locus in LD formation of iMG further strengthens the notion of a causal role of LD in mediating LOAD genetic risk factors, providing potential mechanistic targets for therapeutic development targeting AD.

## Methods

### Human iPSC lines and culture

The human iPSC lines (*n*=62) used for ATAC-seq (Extended Data Table 1) were all derived at Rutgers University Cell and DNA Repository (RUCDR) (also known as NIMH Stem Cell Center and Infinity Biologix, currently Sampled). The human iPSC lines were generated by using the Sendai virus method to ensure it is being integration-free and have undergone the following quality controls (QC): IF staining for pluripotency, mycoplasma contamination test, in-house RNA-seq-based pluripotency test (Pluritest) and eSNP-karyotyping^7,31^ or G-band karyotyping at RUCDR. All donors have European ancestry and were previously used for SZ GWAS studies^72,73^. All donors were also analyzed for copy number variants (CNVs), and none have large CNVs (>100 kb)^74^. There are 29 SZ cases and 33 controls, of which 37 are males with an average age of 49.5 years old (SZ case-control status or age does not affect ASoC mapping^7,31^). Two control-donor human iPSC lines, CD04 and CD09 (abbreviated from the full cell line IDs: CD0000004 and CD0000009), were used in CRISPR/Cas9 editing. Human iPSC were cultured using a feeder-free method on matrigel (Thermofisher)-coated plates in mTeSR plus media (100–0276, StemCell). The media were changed every other day, and cells were passaged as clumps every 4–6 days using ReLeSR (100–0483, StemCell). The NorthShore University HealthSystem institutional review board (IRB) approved this study.

### Human brain samples and gene expression analysis

Frozen human brain samples (frontal pole BA10 region) were received through the NIH biobank from Harvard Brain Tissue Resource Center and the University of Miami Brain Endowment Bank (Extended Data Table 10) and stored at −80°C. For the *PICALM* expression assay, the grey matter was dissected from brain blocks on dry ice. RNA was isolated using a Direct-zol RNA MiniPrep Kit (Zymo), per the manufacturer’s instructions. RNA was reverse-transcribed into complementary DNA using a High-Capacity cDNA Reverse Transcription Kit (Applied Biosystems), per the manufacturer’s instructions. Quantitative PCR (qPCR) reactions were set up using PowerUp SYBR Green Master Mix for qPCR (Applied Biosystems) and run on a QuantStudio Real-Time PCR System (Applied Biosystems). The data were analyzed using 2-ΔΔCt method and normalized to *COTL1*. Primer sequences used *PICALM*-exon1-F: TCTGCCGTATCCAAGACAGT; *PICALM*-exon2-R: AAGACCACCACCCAACTACT; *COTL1*-F: CCAAGATCGACAAAGAGGCTT; *COTL1*-R: CGATGGTGGAGCCGTCATATTT.

### Human brain immunofluorescence

Paraffin sections (5 μm) of post-mortem human brain samples were received from Goizueta Alzheimer’s Disease Research Center at Emory University. Epitope retrieval was performed using Decloaking Buffer (Biocare Medical) at 95°C for 30 min. Antibody staining was conducted at room temperature (RT) using the intelliPATH FLX system and reagents supplied by the manufacturer (Biocare Medical). Nonspecific epitopes were blocked for 20 min, followed by primary antibody incubation for 90 min. After washing, secondary antibodies were incubated for 90 min. Autofluorescence was quenched by treatment with 0.2% Sudan Black B, and nuclei were stained using Hoechst 33342. Primary antibodies used were rabbit anti-PICALM (Sigma: HPA019061 [1:500]) and goat anti-IBA1 (Abcam: ab5076 [1:500]). Coverslips were mounted using VectorShield mounting medium. Images were acquired on an automated Nikon Eclipse Ti2 microscope fitted with the Yokogawa spinning disk field scanning confocal system and Photometrics PRIME 95B sCMOS camera, using 60X objective (0.18 μm/pixel). Z-stack images were deconvolved in Huygens professional (V:23.10) and processed using Fiji/ImageJ2 (V:2.14.0/1.54f).

### Microglia (iMG) differentiation from human iPSC

iMG were generated from human iPSC lines as in our previous study^75^ using the Brownjohn’s method^36^. At least 2 days after passaging, when human iPSC reached confluency ~80%, they were dissociated with Accutase (07920, StemCell) and plated at 10,000 cells per well in 96-well round bottom ultra-low attachment plates (7007, Corning) in 100 μL embryoid body (EB) media (complete mTeSR with 50 ng/ml BMP-4 (120–05, PeproTech), 20 ng/ml SCF (300–07, PeproTech), 50 ng/ml VEGF-121 (100–20A, PeproTech), and ROCK inhibitor (1254/1, R&D Systems). Hematopoietic media was prepared by adding to the X-VIVO 15 (BE08–879H, Lonza), 1% GlutaMax (35050061, Thermofisher), 1% Pen/strep (10378016, Thermofisher), 55 μM β-mercaptoethanol (21985023, Thermofisher), 100 ng/ml M-CSF (300–25, PeproTech), and 25 ng/ml IL-3 (200–03, PeproTech). After 5 days of culturing EBs in hematopoietic media, primitive macrophage progenitors (PMPs) started appearing in the suspension and were produced continuously in suspension for 34 days. After 10 days of culturing EBs, PMPs were harvested from suspension and plated in RPMI 1640 media (21870076, Thermofisher) at 180,000 cells/cm^2^ in 6- or 12-well plates. Complete iMG media: RPMI 1640 with adding 10% FBS (S11150H, R&D Systems), 1% Pen/strep, 1% GlutaMax, 100 ng/ml IL-24 (200–34, PeptroTech), and 10 ng/ml GM-CSF (300–03, PeproTech). The final differentiation of PMPs into iMG occurred over 25 days.

### Astrocyte (iAst) differentiation from iPSC-derived NPCs

NPC were prepared using PSC Neural Induction Medium (A1647801, Thermofisher) following the vendor’s protocol. NPCs were differentiated to astrocytes by seeding dissociated single cells at 15,000 cells/cm^2^ density on matrigel-coated plates in Astrocyte medium [1801, ScienCell: Astrocyte medium, 2% FBS (0010), astrocyte growth supplement (1852), and 10 U/ml Pen/Strep solution (0503)]. Initial NPC seeding density and single-cell dissociation are critical, particularly during the first 30 days of differentiation, to efficiently generate a homogenous population of astrocytes. On day −1, NPCs were pipetted with a p1000 pipette 3–5 times to yield a single-cell suspension and inhibit cell death. The NPC medium was switched to the Astrocyte medium at day 0. From day 2, cells were fed every 48 hr for 20–30 days. After 30 days of differentiation, astrocytes were split 1:3 weekly with Accutase and expanded up to 120 days (15–17 passages) in the Astrocyte medium. The final differentiation of iAst occurred over 30 days.

### Differentiation of glutamatergic neurons

We followed an established protocol to differentiate iPSC into glutamatergic neurons (iN-Glut)^76^. In brief, iPSCs were dissociated into single cells by Accutase (07920, StemCell) and replated at 7.5 × 10^5^ cells per well in a 6-well plate using mTeSR plus media (100–0276, StemCell) with 5 μM ROCK inhibitor (1254/1, R&D Systems) on Day (−1). On Day 0, cells were infected by 200 μl/well lentivirus cocktail containing 100 μl NGN2 virus and 100 μl rtTA virus^76^. After a two-day puromycin selection, iGluts On Day 5 were dissociated with Accutase and plated as a 100 μl blob on glass coverslips (GG-12–15-Pre, Neuvitro). From day 6, 500 μl of neuronal culture media were added into each well with a half-volume of medium change every 3 days for continuous culturing. Doxycycline was withdrawn on Day 21 of differentiation. The final differentiation of iNs occurred over 30 days.

### Differentiation of dopaminergic neurons

The protocol for the differentiation of dopaminergic neurons (iDN) was adapted from Gonzalez *et al*.^77^. Briefly, dopaminergic priming media was added to the cells at 50% confluence on Day 0. On Day 7, the cells were replated onto 6 well plates coated with matrigel at 5 × 10^5^ cells/well and switched to Dopaminergic differentiation media. Media was changed every other day. On Day 30, dopaminergic neurons were harvested using Accutase (07920, StemCell) for ATAC-seq.

### Differentiation of GABAergic neurons

We generated GABAergic neurons (iN-GA) from NPCs using the protocol from Yang *et al*.^78^ but with NPCs as source cells. NPCs were single-cell at 200,000 cells/cm^2^ on Day 0. Virus-Neural Expansion Medium Cocktail was added on Day 1 with ASCL1-puro and DLX2-hygro virus and replaced by the Expansion Medium Cocktail with 2 μg/ml doxycycline (D9891, Sigma) the same day. Puromycin and Hygromycin selection were conducted between day 2 to day 6. On day 7, we switched media to conditioned NeuralbasalPlus Media and changed media every 3 days. Doxycycline was withdrawn on Day 16, and 50 nM Ara-C was included in the media if non-neuron cells were observed. On Day 28, neurons were harvested using Accutase for ATAC-seq.

### Immunofluorescence staining of iMG and iAst

For characterization of iMG and iAst, cells were fixed in 4% PFA (P6148, Sigma) for 10 min at RT. Cells were incubated with primary antibodies at 4°C overnight in 3% BSA containing 0.3 % Triton X-100, followed by 3 washes in PBS for 5 min each. Cells were then incubated with secondary antibodies at RT for 1 hr in 3% BSA containing 0.3 % Triton X-100. After which, cells were washed another 3 times with PBS and incubated in 0.5 μg/ml DAPI (4′, 6-diamidino-2-phenylindole) at RT for 10 min. The images were acquired using a Nikon ECLIPSE TE2000-U microscope.

Primary antibodies used for microglia immunofluorescence and their dilutions for incubation were rat anti-TREM2 (MABN755, Sigma, 1:100), rabbit anti-CD45 (SAB4502541, Sigma, 1:200), mouse anti-PU1 (MAB114, R&D Systems, 1:100), mouse anti-IBA1 (MA5–27726, Thermofisher, 1:100), rabbit anti-ATP6AP2 (SAB2702080, Sigma, 1:100), rabbit anti-VAMP1 (702787, Thermofisher, 1:100), rabbit anti-HMGCR (SAB4200528, Sigma, 1:100), and mouse anti-CD74 (14-0747-82, Thermofisher, 1:100). Secondary antibodies were Alexa 488 donkey anti-rat (A21208, Invitrogen, 1:1000), Alexa 594 anti-rabbit (A21207, Invitrogen, 1:1000), Alexa 647 anti-mouse (A32787, Invitrogen, 1:1000), Alexa donkey 594 anti-mouse (A21203, Invitrogen, 1:1000), and Alexa donkey 647 anti-rabbit (A32795, Invitrogen, 1:1000). Primary antibodies used for astrocyte (iAst) immunofluorescence and their dilutions for incubation were rabbit anti-Vimentin (3932, Cell Signaling, 1:200), mouse anti-GFAP (G3893, Sigma, 1:100), and mouse anti-s100β (S2532, Sigma, 1:100). Secondary antibodies were Alexa 488 donkey anti-rabbit (A21206, Invitrogen, 1:1000) and Alexa 594 anti-mouse (A21203, Invitrogen, 1:1000).

### RNA isolation and sequencing

Cells from human iPSC, iAst and iMG cultures were dissociated with Accutase (07920, StemCell) and total RNA was extracted with the RNeasy Plus Kit (74134, Qiagen). cDNA was reverse transcribed from RNA with a high-capacity cDNA reverse transcription kit (4368814, Applied Biosystems). Subsequent RNA-seq was performed by Novogene on the Illumina NovaSeq 2000 platform with targeted 30 M paired-end reads (2 × 150 bp) per sample.

### RNA-seq data and DE analyses

Raw FASTQ files were aligned to human hg38 genome GRCh38.p14 using STAR v2.7.2 and counted according to GENCODE annotation release version 35 on the fly. The ComBat-seq function from R package sva was applied to eliminate any potential batch-derived bias prior to EdgeR analysis. For EdgeR-based differential gene expression analysis, the general linear models (glmQLFit and glmQLFtest) were used with design matrices, including cell line information as coefficients to remove line-specific effects. DE significant genes were defined as BH-adjusted *P* value (FDR) < 0.05 (same as the default value defined in topTags). Counts per million (CPM) values were used for PCA analysis and graph plotting.

### ATAC-seq

ATAC-seq sample preparation was performed as previously described^7,31^. Briefly, 75,000 viable cells were used for each transposition mixture reaction. Samples were then incubated at 37°C for 30 min on a thermomixer at 1,000 rpm. The eluted DNA was shipped to the University of Minnesota Genomic Center for library preparation and ATAC-seq.

### ATAC-seq data analysis and peaking calling

All raw sequence reads generated by Illumina NextSeq were demultiplexed at the University of Minnesota Genomics Center and provided as 2×75 bp paired-end FASTQ files (targeting 60 M reads per sample). Only paired-end reads that survived Trimmomatic processing v0.39 (ILLUMINACLIP:NexteraPE-PE.fa:2:30:7, SLIDINGWINDOW:3:18, MINLENGTH:26) were retained. The FASTQ files were individually mapped against the human genome reference file including decoy sequences (GRCh38p7.13/hg38, 1000 Genome Project) using bowtie2 (-x 2000, -mm --qc-filter –met 1 –sensitive –no-mixed -t) and subsequently merged and sorted as BAM-formatted files using samtools v1.14, with only uniquely high-quality mapped reads (MAPQ > 30, SAM flags 0×1, 0×2) retained. Picard tools MarkDuplicate was then used to remove all PCR and optical duplicated reads from the BAM file.

To further eliminate allelic bias towards reference alleles during the aligning step, we performed WASP calibration on the generated raw BAM files^79^. Briefly, we first called the VCF file profiles on all SNP variants per sample individually using GATK HaplotypeCaller to generate cell line-specific VCF files. The cell line-specific VCFs were used as the basis of WASP calibration and re-alignment, and new WASP-calibrated BAM file sets were collected as the final output for the following peak and ASoC SNP calling. All analyzed ATAC-seq samples passed standard QC based on the characteristic nucleosomal periodicity of the insert fragment size distribution and high signal-to-noise ratio around transcription start sites (TSS).

### Allele-specific open chromatin (ASoC) mapping

MACS2^38^ was used to generate peak files (narrowPeak format) with recommended settings at FDR = 0.05 (-f BAMPE, --nomodel, --call-summits --keep-dup-all -B). Peaks that fell within the ENCODE blacklisted regions were removed. Also, we removed peaks falling within chromosomes X, Y, mitochondrial genome, and decoy regions.

GATK (version 4.1.8.1) was used for ASoC SNP calling, as recommended by the GATK Best Practices (software.broadinstitute.org/Gatk/best-practices/)^80^. As noted above, WASP-calibrated BAM files (without sub-sampling) generated from the ATAC-seq pipeline were used as input and variants were called against human GRCh38.p14 (hg38) genome and the corresponding dbSNP version 154, and only reads with MAPQ score ≥30 were used (-stand_call_conf 30). Subsequently, recalibration of SNPs and Indels was performed in tandem using the VariantRecalibrator function (-an DP -an QD -an FS -an SOR -an MQ -an ReadPosRank - Sum mode SNP -tranche 100.0 -tranche 99.5 -tranche 95.0 -tranche 90.0) and scores recalibrated using reference database including HapMap v3.3 (priority = 15), 1000G_omni v2.5 (priority = 12), Broad Institute 1000G high confidence SNP list phase 1 (priority = 10), Mills 1000G golden standard INDEL list (priority = 12), and dbSNP v154 (priority = 2). Heterozygous SNP sites with tranche level >99.5% were extracted. To reduce bias introduced by any acquired (or “*de novo*”) mutations during cell growth, only SNPs with corresponding rs# records found in dbSNP v154 were retained. Biallelic SNP sites (GT: 0/1) with minimum read depth count (DP) ≥ 20 and minimum reference or alternative allele count ≥ 2 were retained. The binomial *p*-values (non-hyperbolic) were calculated using the binom.test(x, n, P = 0.5, alternative = “two.sided”, conf.level = 0.95) from the R package, and Benjamini & Hochberg correction was applied to all qualified SNPs as the correcting factor of R function p.adjust(x, method = “fdr”). We set the threshold of ASoC SNP at FDR value = 0.05.

The read pileup statistics proximal to SNP sites were generated using samtools mpileup function, and differential of allele-specific reads was performed using the SNPsplit Perl package (www.bioinformatics.babraham.ac.uk/projects/SNPsplit/). The final readouts from both read pileup and SNP-specific reads were visualized using the R package Gviz. In addition, when comparing the changes in chromatin accessibility caused by genotypes across samples or between different cell types, read counts were scaled and normalized using the deepTools package bamCoverage function and re-scaled to reads per genomic content (RPGC) as the base unit^81^. We confirmed no obvious mapping bias to reference alleles by visualizing the volcano plots that graph the allelic read-depth ratios against -log_2_*p*-values in scatter plots (Supplementary Fig. 2a).

### Stratified linkage disequilibrium score regression (sLDSC) for GWAS enrichment analysis

sLDSC^82^ analysis were performed by using the hg38 version of European genotype data (SNPs) from 1000 Genomes Phase 3 and v2.2 baseline linkage disequilibrium/weights as previously described^7^. Briefly, linkage disequilibrium score estimations were pre-calculated from the hg38 version of the 1000 Genomes EUR file set (w_hm3_no_hla.snplist), window size 1 cM (ld-wind-cm 1). We used the summary statistics of major psychiatric disorders and non-psychiatric diseases (Extended Data Table 8) for partition heritability, with several data sets lifted over from hg19 to hg38 when necessary. Disease-specific regressions were performed using hm3 SNP weights against each disease independently for cell type-specific analysis.

### Torus GWAS enrichment analysis

Bayesian hierarchical model (TORUS) was applied to perform an SNP-based enrichment analysis to explore whether ASoC SNPs are enriched in any of the diseases^83^ as previously described^7^. For the GWAS enrichment test, ASoC SNPs derived from each cell type were applied independently. The annotations are encoded as Boolean (true if an SNP has an annotation). The GWAS datasets used for enrichment/TORUS analysis were consistent with the diseases analyzed in sLDSC. Univariate analysis was performed to assess the enrichment of ASoC SNPs in each GWAS dataset.

### CRISPR/Cas9 editing of human iPSC

CRISPR guide RNA (gRNA) sequences were designed using an online tool (crispr.mit.edu), and we selected the gRNAs with the highest score (specificity) (Extended Data Table 16). The gRNAs were cloned into pSpCas9(BB)-2A-Puro vector (Addgene #62988) for co-expression with Cas9 based on an established protocol^84^. For transfection, 3 μg of CRISPR/Cas9-gRNA construct was combined with 3 μg ssODNs (1:1 ratio) in Opti-MEM media (31985062, Thermofisher) and Lipofectamine stem reagent (STEM00001, Thermofisher) was used for transfection. Puromycin selection was performed to eliminate untransfected cells and was withdrawn after 72 hr of transfection. Resistant colonies were collected 14 days after transfection and a small amount of DNA from each colony was used for Sanger sequencing to verify editing. Pure clone was confirmed for on-target editing and absence of off-target editing (see below).

### Quality control of the CRISPR-edited human iPSC lines

Primers were designed to amplify regions corresponding to the 4 top-ranking predicted off-targets for checking on-target and off-target editing. All primer and oligo sequences were listed in Extended Data Table 16.

To confirm the pluripotency of CRISPR-Cas9 edited human iPSC lines, the cells were stained against pluripotency markers including rabbit anti-Oct-4 (ab181557, Abcam, 1:250), goat anti-NANOG (AF1997-SP, R&D Systems, 1:50), mouse anti-SSEA4 (ab16287, Abcam, 1:250). Images were taken with a Nikon ECLIPSE TE2000-U microscope.

eSNP-karyotyping was performed for all cell lines used to eliminate possible chromosomal abnormalities, as previously described^7,31^. RNA-seq data were processed by the eSNP Karyotyping package^85^ rewritten for GATK 4 and R 4.2 using raw FASTQ files as the input. Alignment to the human hg38 genome was performed by Bowtie2 v2.5.1, and only common SNPs (MAF > 0.05) from dbSNP 154 were retained for zygosity block analysis. The plotted zygosity block size was 1.0 Mb.

### CRISPRoff epigenome editing of human iPSC

CRISPRoff guide RNA (gRNA) sequences were designed using online tool (benchling.com) (Extended Data Table 16). The gRNAs were cloned into CROPseq-Guide-Puro vector (Addgene #86708) for co-expression with CRISPRoff-v2.1 (Addgene #167981) based on an established protocol^86^. After 72 hr of drug selection, transfection cells were sorted using a BD FACSAria II and the sorted cells were passaged two times and then differentiated into iMG.

### Gene expression analysis by qPCR

For qPCR, reverse transcription was performed using a ThermoFisher High-capacity RNA-to-cDNA reverse transcription kit (4366596, Applied Biosystems) with random hexamers according to the manufacturer’s protocol. qPCR was performed using TaqMan Universal PCR Master Mix (4364338, Applied Biosystems) on a Roche 480 II instrument, using gene-specific FAM-labelled TaqMan probes or custom-designed probes from IDT (Extended Data Table 16). GAPDH was used as the control.

### Myelin isolation from mouse brains for phagocytosis

Myelin was isolated from mouse brains by homogenization in 0.32 M Sucrose Buffer (0.32 M sucrose and 2 mM EGTA). Samples were then further homogenized using a Dounce homogenizer, layered on top of 0.85 M Sucrose Buffer (0.85 M sucrose and 2 mM EGTA), and centrifuged at 75,000 × g at 4°C for 30 min. Crude myelin was collected from the interface, resuspended in Tris-Cl Buffer (0.2 M Tris-HCl, pH7.5), and homogenized using a Dounce homogenizer. Samples were centrifuged at 75,000 × g at 4°C for 15 min. Pellets were resuspended in Tris-HCl Solution (20 mM Tris-HCl, 2 mM EDTA, 1 mM DTT, pH 7.5) and homogenized using a Dounce homogenizer. Samples were centrifuged at 12,000 × g at 4°C for 15 min, and pellets were resuspended in Tris-Cl Solution. Samples were then centrifuged at 12,000 × g at 4°C for 10 min. Pellets were resuspended in 0.32 M Sucrose Buffer, layered on top of 0.85 M Sucrose Buffer, and centrifuged at 75,000 × g at 4°C for 30 min. Purified myelin was collected from the interface, resuspended in Tris-HCl Buffer, and homogenized using a Dounce homogenizer. Samples were centrifuged at 75,000 × g at 4°C for 15 min, and the pellets were resuspended in Tris-HCl solution and homogenized using a Dounce homogenizer. Samples were centrifuged at 12,000 × g at 4°C for 15 min, pellets resuspended in Tris-HCl solution and centrifuged at 12,000 × g at 4°C for 10 min. Pellets were resuspended in Tris-HCl Solution, aliquoted, and stored at −80°C. The protein content of isolated myelin was determined by a BCA Protein Assay Kit.

### Phagocytosis assay for iMG

iMG were grown on MatTek 96 well plate with glass bottom (NC1844174, Fisher Scientific) until Day 25. For Aβ phagocytosis, the beta-amyloid (1–42) aggregation kit was used (A-1170–025, rPeptide). The peptide was resuspended in 5 mM Tris at 1 mg/ml concentration. Myelin and Aβ peptides were labeled using the pHrodo Red Microscale Labeling Kit (P35363, Thermofisher) following the vendor’s protocol.

For pHrodo phagocytosis experiment pHrodo-labeled myelin or Aβ was diluted to 15 μg/ml in RPMI 1640 media (21870076, Thermofisher), bath sonicated for 1 min and added to the iMG cells along with CellMask Green Plasma Membrane Stain (C37608, Thermofisher, 1:1000) and NucBlue Live ReadyProbes Reagent (R37605, Thermofisher, 2 drops per ml), mixed gently and incubated at 5% CO_2_, 37°C for 30 min. As a negative control, 10 μM Cytochalasin D (PHZ1063, Thermofisher) was added to cells along with pHrodo-labelled protein and retained throughout uptake assays. Live Imaging (5% CO_2_, 37°C) was performed for a total of 3 hr using a Nikon ECLIPSE TE2000-U microscope at 45 min intervals.

For pHrodo phagocytosis experiment that included LD staining, the iMG cells were treated with 1 μM Triacsin C (TrC, 10007448, Cayman Chemical) in complete iMG media for 18 hr. Next, BODIPY 493/503 (D3922, Thermofisher, 1:1000) and CD45 antibodies (14-0451-82, Thermofisher, 1:500) were added to cells with and without TrC and incubated for 30 min and quickly washed 2 times with RPMI 1640. Then pHrodo-labeled myelin or Aβ was diluted to 15 μg/ml in RPMI 1640 media, bath sonicated for 1 min and added to the iMG cells along with NucBlue Live ReadyProbes Reagent and incubated at 5% CO_2_, 37°C for 30 min. As a negative control, 10 μM Cytochalasin D was added to cells along with pHrodo-labelled protein and retained throughout uptake assays. Live Imaging (5% CO2, 37°C) was performed for a total of 3 hr using Nikon ECLIPSE TE2000-U microscope at 45 min intervals. FiJi/ImageJ software was used to quantify pHrodo fluorescence intensity (fiji.sc).

### Chromatin immunoprecipitation (ChIP)

ChIP qPCR assay was performed by combining two protocols from Simple ChIP Enzymatic Chromatin IP Kit (91820, Cell Signaling) and Magna ChIP A/G Chromatin Immunoprecipitation Kit (17–10085, Sigma). 10^7^ cells were used for each reaction with 1% formaldehyde (28908, Thermofisher) crosslinking in 20 ml of cell suspension. The Cell Signaling IP Kit was used for nuclei preparation and subsequent recovery reactions according to the vendor’s protocols. For chromatin digestion, 1.25 μl of Micrococcal Nuclease was used and incubated for 20 min at 37°C to digest DNA to the length of approximately 150–900 bp. To break the nuclear membrane, lysate was sonicated for 3 sets of 20-sec pulses with a 1/8-inch probe.

The Sigma ChIP Assay Kit was used for the reaction according to the vendor’s instructions. Normal rabbit IgG (#2729, Cell Signaling) was used as negative control. 1 μl of Proteinase K was used for reverse cross-linking of Protein/DNA complexes to free DNA at 62°C for 2 hr with shaking, followed by incubation at 95°C for 10 min. For each reaction, DNA was eluted into 50 μl of Elution Buffer “C”. qPCR was performed using TaqMan Universal PCR Master Mix (4364338, Applied Biosystems) on a Roche 480 II instrument, using IDT custom probe for detection PICALM/PU.1 ratio. The same DNA product was also subjected to Sanger sequencing for the heterozygous site rs10792832 sequencing.

### Fatty acid (Red-C12) transfer assay for iMG

Microglia (iMG) and astrocytes (iAs) were grown on coverslips until Day 25. Neurons (iNs) were grown on coverslips until Day 30. Cells were incubated with 8 μM BODIPY 558/568 (Red-C12, D3835, Thermofisher) for 16 hr in neuronal growth media, washed twice with warm PBS, and incubated with fresh media for 1 hr. Red-C12 labelled neurons and unlabelled astrocytes/microglia were washed twice with warm PBS, RedC12 intensity was examined by fluorescence microscopy.

### LD staining with BODIPY for iMG

iMG were grown on glass coverslips until Day 25. Cells were then fixed for 30 min at RT with 4% PFA (P6148, Sigma) in PBS, briefly washed in PBS two times and incubated in PBS with BODIPY 493/503 (D3922, Thermofisher, 1:1000 from 1 mg/ml stock solution in DMSO) and DAPI for 10 min at RT. BODIPY intensity was examined by fluorescence microscopy.

### LD staining with LipidTOX for iMG

iMG were grown on glass coverslips until Day 25. Cells were then fixed for 30 min at RT with 4% PFA (P6148, Sigma) in PBS, briefly washed in PBS two times and incubated in PBS with LipidTOX (H34476, Thermofisher, 1:1000) and DAPI for 1 hr at RT. LipidTOX intensity was examined by fluorescence microscopy.

### Reactive Oxygen Species (ROS) staining for iMG

iMG were grown on glass coverslips until Day 25. Cells were treated with 1μM Triacsin C (TrC, 10007448, Cayman Chemical) in complete iMG media for 18 hr. Cells were subsequently incubated in complete iMG media with CellROX Deep Red (C10422, Invitrogen, 1:500) for 30 min at 37°C. After that, the cells were stained with BODIPY according to described protocol (lipid droplets staining with BODIPY) to detect LD. CellROX intensity was examined by fluorescence microscopy.

### LD staining with lipid peroxidation sensor BODIPY C11 for iMG

iMG were grown on glass coverslips until Day 25. Cells were treated with 1μM Triacsin C as described in ROS staining. Next, cells were incubated in complete iMG media with BODIPY 581/591 C11 (D3861, Thermofisher, 1:1000) for 15 min at 37°C, then fixed for antibody staining by rat anti-TREM-2 (MABN755, Sigma, 1:100). 568 nm excitation wavelength was applied to excite reduced BODIPYC11 and 488 nm excited oxidized BODIPYC11. FiJi/ImageJ software was used to quantify BODIPYC11 fluorescence intensity (fiji.sc).

### Filipin staining for iMG

iMG were grown on glass coverslips until Day 25. Cells were then fixed for 10 min at RT with 4% PFA (P6148, Sigma) in PBS. Cells were incubated with a solution of filipin (0.1 mg/ml, F‐9765 Sigma) for 30 min. After staining with were rat anti-TREM-2 (MABN755, Sigma, 1:100), cells were washed and counterstained with propidium iodide (0.35 μg/ml; P4170, Sigma) for 10 min at RT. 405 nm excitation wavelength was used to excite filipin. For each field of view, filipin fluorescence intensity was calculated by dividing the number of blue puncta by the number of microglia. Then, values were normalized to the filipin fluorescence intensity value of the risk allele and used for statistical analysis. The Fiji software was used In all experiments involving fluorescence quantification (fiji.sc).

### C20 cell culture

C20 cells were maintained in DMEM/F12 media containing 10% fetal bovine serum (FBS) and 1% penicillin/streptomycin. *PICALM* exon 1 was targeted using the following oligonucleotide sequences – sgRNA F: CACCGgccggtgacactgtgctggg and R: AAACcccagcacagtgtcaccggcC. Control oligo sequences were generated using sequences not specific to the human genome. Recombinant lentiviruses were generated in HEK293T cells using MISSION Lentiviral Packaging Mix (Sigma: SHP001). C20 cells were transduced with filtered virus-containing media, and stable pools were selected in blasticidin (20 μg/mL).

### Immunoblots for C20 cells

Cells were lysed in RIPA buffer (50 mM Tris, 150 mM NaCl, 0.5% sodium deoxycholate, 1% Triton X-100, 0.1% SDS, 5 mM EDTA, pH 8) containing complete protease inhibitors (Roche), sonicated, and lysates cleared by centrifugation (21,000 × g for 2 min). 50 μg of lysate was used per lane on 4–20% SDS PAGE gels and transferred onto nitrocellulose membranes. Non-specific sites were blocked with PBS containing 1% BSA and 1% fish gelatin at RT for 1 hr. Membranes were incubated with primary antibodies rabbit anti-PICALM (Sigma: HPA019061 [1:500]) and mouse anti-β-actin (Proteintech: 66009–1-lg [1:50,000]) in at 4°C for 16 hr. Secondary antibodies IRDye 680RD donkey anti-rabbit IgG and IRDye 800CW donkey anti-mouse IgG (Li-COR) were incubated at RT for 2 hr. Blots were imaged and quantified on a Li-COR Odyssey infrared imaging system.

### Myelin phagocytosis for C20 cells

Myelin isolated from the mouse brain (as described above) was conjugated to pHrodo-Green (Thermo Fisher: 35369) or pHrodo-Red (Thermo Fisher: P36600), per the manufacturer’s instructions. C20 cells were incubated with pHrodo-conjugated myelin (20 μg/mL) at 37°C (5% CO_2_) for 1 hr. Negative controls were treated with cytochalasin D (10 nM; Invitrogen). After fixation, cells were stained with rabbit anti-BIN1 antibody (Proteintech: 14647–1-AP [1:500]) for 2 hr. Images were acquired on a Nikon Eclipse Ti2 (Yokogawa spinning disk field scanning confocal) microscope at 20× magnification and captured using a Photometrics PRIME 95B sCMOS camera. Single-plane images were processed using FiJi/ImageJ software to threshold whole-cell masks (created from BIN1 staining). The integrated density of pHrodo-myelin within each cell was measured from five random fields of view (per biological replicate), and the median values from each replicate were used for statistical analysis.

### LD staining for C20 cells

Live C20 cells were labeled with BODIPY (2 μM in PBS) at 37°C (5% CO_2_) for 15 min. All remaining steps were performed at RT. Cells were fixed with 4% PFA for 30 min, and nuclei were labeled with Hoechst 33342. The volumes of BODIPY positive droplets and nuclei numbers were quantified from deconvolved image stacks using ImageJ/Fiji software. Five random fields of view (per biological replicate) were acquired for quantification, and the median values from each biological replicate were used for statistical analysis.

### Organelle marker staining for C20 cells

C20 cells were fixed with 4% PFA and blocked (3% BSA, 50 mM NH4Cl, 10 mM glycine, PBS) at RT for 30 min primary antibodies. For transferrin endocytosis, C20 cells were washed and incubated in uptake media (DMEM containing 25 mM HEPES) for 1 hr. Cells were treated with 10 μg/mL transferrin Alexa Fluor 555 conjugate (Invitrogen: T35352) in DMEM/HEPES containing 1 mg/mL BSA at 37°C for 30 min. Cells were then chilled to 4°C, and non-internalized transferrin was washed from cell surfaces with acid wash (0.5 M NaCl, 0.2 M acetic acid, pH 2.8) before fixing the cells in PFA. For cholera toxin internalization, cells were washed with labelling media (1 mg/mL BSA in serum-free DMEM containing 25 mM HEPES), chilled to 4°C, and treated with 100 nM cholera toxin subunit B Alexa Fluor 647 conjugate (Invitrogen: C34778) in labelling media at 4°C for 10 min. Cells were then washed with PBS and incubated in labelling media at 37°C (5% CO_2_) for 45 min. Cells were washed with ice-cold PBS and fixed with 4% PFA (in PBS) at room temperature for 15 min. Nuclei were labelled with Hoechst 33342.

For Giantin, EEA1, and AP-4 staining, cells were treated with rabbit anti-Giantin (Covance: PRB-114C [1:5,000]), mouse anti-EEA1 (BD Transduction Laboratories: 610457 [1:500]) or mouse anti-AP44 (adaptin-e) antibody (BD Transduction: 612018 [1:100]) at RT for 2 hr. All C20 cell mages were acquired on a Nikon Eclipse Ti2 spinning disk field scanning confocal microscope at 60× magnification. Z-stacks (100 nm step size) were processed to generate maximum intensity projections using FiJi/ImageJ.

### Imaging quantification and statistical analyses

For imaging analyses of phagocytosis, LD (BODIPY or LipidTox staining), CellRox, RedC12 transfer, and BODIPY C11 staining, we assayed fluorescence intensity, puncta density, puncta area, and/or puncta size as specified in the corresponding Figures from 2–3 biological replicates (independent cell culture wells or coverslips) each with various number of fields of views (FOV). For each FOV, fluorescence intensity was calculated by dividing the number of fluorescent puncta by the number of microglia. The number of puncta and total area of puncta were acquired by applying a threshold to the respective fluorescent areas and performing the “Analyze particles” function. The number of microglia was acquired by applying a threshold to the DAPI fluorescent area and measuring a number of nuclei through the same function. The images were acquired in a way that sample and genotype identification were unknown to the operator. In all experiments, Student’s t-test (two-tailed, unpaired, heteroscedastic) was used unless otherwise specified. Data analysis was performed using R 4.3.2, GraphPad Prism 9, and Microsoft Excel. Results were considered as significant if *p* < 0.05 (*: *P* < 0.05; **: *P* < 0.01; ***: *P* < 0.001). All data with error bars were presented as mean ± SEM. For ATAC-seq analysis of ASoC SNPs, the binomial test in R package was used for testing allelic bias and Benjamini-Hochberg correction was applied to all qualified ASoC SNPs. For RNA-seq gene DE analysis, limma/EdgeR using the generalised linear model (GLM) F test was used, and the Benjamini-Hochberg procedure was used to adjust *p*-values accounting for multiple testing.

## Extended Data

**Extended Data Fig. 7.**
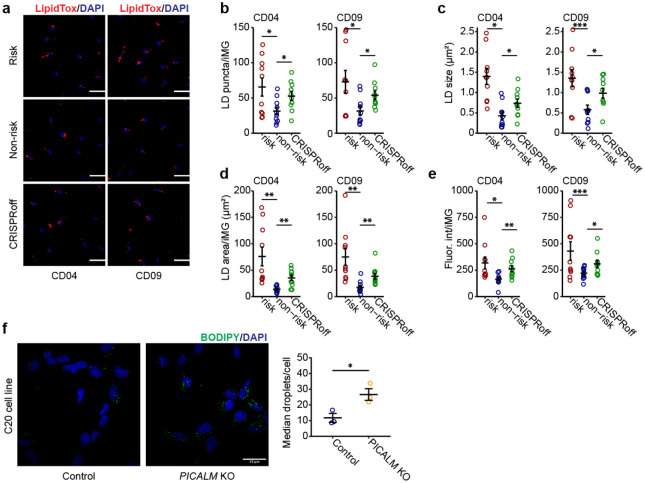
Fluorescence staining of LD using LipidTox in iMG carrying LOAD risk or non-risk allele and in iMG with *PICALM* CRISPR-off (a) to (e) and LD staining in C20 *PICALM*-KO cells (f). (a) LD staining with LipidTox. Scale bar, 50 μm. (b) to (e) Increased LD density, size, area, and fluorescence intensity in iMG carrying the LOAD risk allele (vs. non-risk). *PICALM* CRISPR-off reduces LD density, size, area, and fluorescence intensity (vs. non-risk allele). Data are from 2–3 independent wells of 3–4 FOVs each. Student’s *t*-test (2-tailed, heteroscedastic) was used; * *P*<0.05, ** *P*<0.01, ***, *P*<0.001; error bar, SEM. (f) LD (BODIPY^+^) staining in *PICALM*-KO C20 cells (left two panels) and quantification of LD number per cell (right). Each dot represents the median number of droplets per cell for each biological replicate (coverslip). Data are from 3 biological replicates of 5 FOVs each. Student’s *t*-test (2-tailed, heteroscedastic) was used; * *P*<0.05, ** *P*<0.01, ***, *P*<0.001; error bar, SEM.

**Extended Data Fig. 8.**
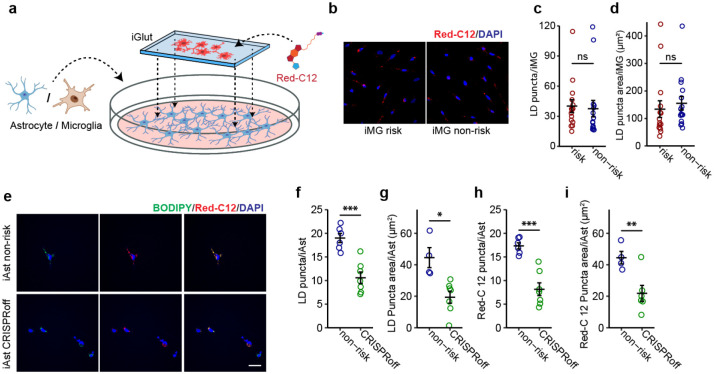
PICALM affects lipid transfer from neurons to iMG and iAst differently. (a) Diagram of the lipid (Red-C12 is a fatty acid analogue) transfer system between neurons (iN-Glut) and iMG or iAst. iN-Glut was labelled with Red-C12 for 18 hr, then co-cultured with iMG or iAst for 4 hr before staining iMG or iAst with BODIPY. (b) Red-C12 imaging in the co-cultured iMG carrying LOAD risk allele or non-risk allele. Scale bar, 50 μm. (c) and (d) Quantification of the transferred Red-C12 area and density in iMG. Data are from line CD04, 3 independent wells of 3–4 FOVs each. Student’s *t*-test (2-tailed, heteroscedastic) was used; * *P*<0.05, ** *P*<0.01, ***, *P*<0.001; error bar, SEM. (e) Red-C12 imaging and LD (BODIPY^+^) staining of the co-cultured iAst with *PICALM* CRISPRoff (over the genetic background of non-risk allele since the LOAD risk allele of *PICALM* does not affect its expression in iAst). Note the partial overlap of Red-C12 and BODIPY staining, suggesting the transferred Red-C12 partially contribute to LD formation in iAst. Scale bar, 50 μm. (f) and (g) Quantifying LD density and area in the co-cultured iAst. (h) and (i) Quantifying the Red-C12 density and area in iAst. Data are from line CD04, 3 independent wells of 3–4 FOVs each. Student’s *t*-test (2-tailed, heteroscedastic) was used; * *P*<0.05, ** *P*<0.01, ***, *P*<0.001; error bar, SEM.

**Extended Data Fig. 9.**
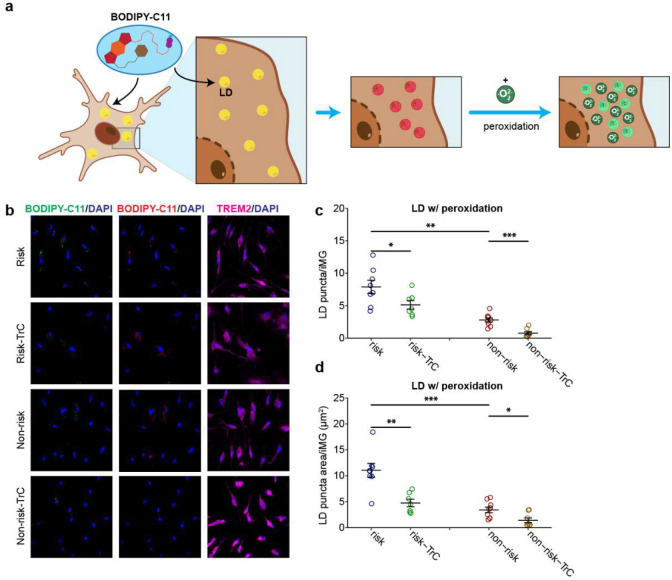
iMG carrying the LOAD risk allele of rs10792832 shows increased lipid peroxidation. (a) A diagram shows C11-BODIPY (581/591), a fluorescent lipid peroxidation sensor, shifts its fluorescence from red to green in the presence of ROS. (b) Fluorescence staining with peroxidation sensor C11-BODIPY in iMG (TREM2^+^) carrying the LOAD risk or non-risk allele in the presence or absence of TrC. Scale bar: 50 μm. (c) and (d) Increased LD (C11-BODIPY green fluorescence) density and area in iMG carrying the LOAD risk allele (vs. non-risk). Note the peroxidized LD density and area are reduced in iMG treated with TrC. 2–3 independent wells of 2–3 FOVs each. Student’s *t*-test (2-tailed, heteroscedastic) was used; * *P*<0.05 ** *P*<0.01, ***, *P*<0.001, ns = not significant; error bar, SEM.

**Extended Data Fig. 10.**
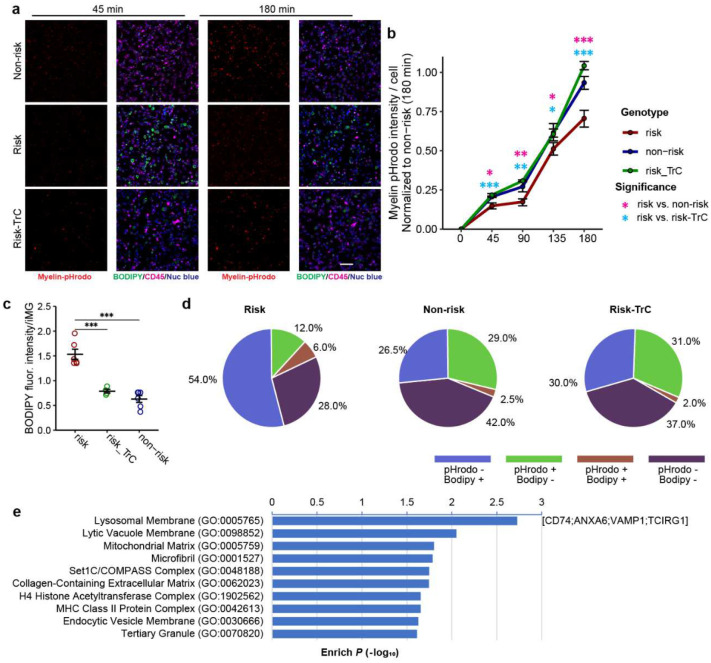
LD accumulation impairs iMG phagocytosis of myelin. (a) Representative fluorescence images show time-dependent phagocytosis of myelin-pHrodo and LD accumulation (BODIPY^+^) in iMG (CD45^+^) carrying the LOAD risk allele or non-risk allele of *PICALM* in the presence or absence of TrC. Scale bar: 100 μm. (b) The reduced myelin-pHrodo intensity in iMG carrying the LOAD risk allele (vs. non-risk) can be rescued by TrC treatment. (c) TrC treatment rescues the LD accumulation in iMG, carrying the LOAD risk allele to a level similar to that of MG, which carries the non-risk allele. (d) Pie charts show the proportion of iMG (CD45^+^) stained positive for myelin-pHrodo, BODIPY, or both from co-localization analysis of the fluorescence images in (a). Note the myelin-pHrodo^+^/BODIPY^+^ iMG are rare in each type of iMG, and TrC treatment rescues the phagocytosis deficit in iMG carrying the LOAD risk allele by mainly converting BODIPY^+^ iMG to phagocytic cells without LD (myelin-pHrodo^+^/BODIPY^−^). Data are from 2 replicate wells of 2–4 FOVs each. Student’s *t*-test (2-tailed, heteroscedastic) was applied; * *P*<0.05, ** *P*<0.01, **, *P*<0.001; error bar, SEM. (e) GO-term enrichment among 30 genes (in Supplementary Fig. 5d) that show the same directional change of expression in iMG carrying the LOAD risk allele of *PICALM* (vs. non-risk) and in mouse LDAM with high LD (vs. low LD). Note that the most enriched GO term is the lysosomal membrane (GO:0005765).

## Supplementary Material

1

## Data Availability

ATAC-seq data for iMG, iAst, and the newly processed NGN2-Glut (*n*=16), GABA (*n*=22) and DN (*n*=31) neurons are accessible at Gene Expression Omnibus under accession code GSE263804. Previously processed ATAC-seq data for NGN2-Glut (*n*=20), GABA (*n*=8), and DN (*n*=8) neurons are accessible at Gene Expression Omnibus under accession code GSE188941. iMG RNA-seq data are available in Gene Expression Omnibus under accession code GSE263809.
