# Supplementary material for: Alzheimer’s disease risk allele of PICALM causes detrimental lipid droplets in microglia
Source: Res Sq. 2024 May 24:rs.3.rs-4407146. Preprint. [Version 1] doi: 10.21203/rs.3.rs-4407146/v1 (PMC11142308; doi:10.21203/rs.3.rs-4407146/v1)
Supplement: 1 [file NIHPPRS4407146V1-supplement-1.pdf]

## Supplementary Figures

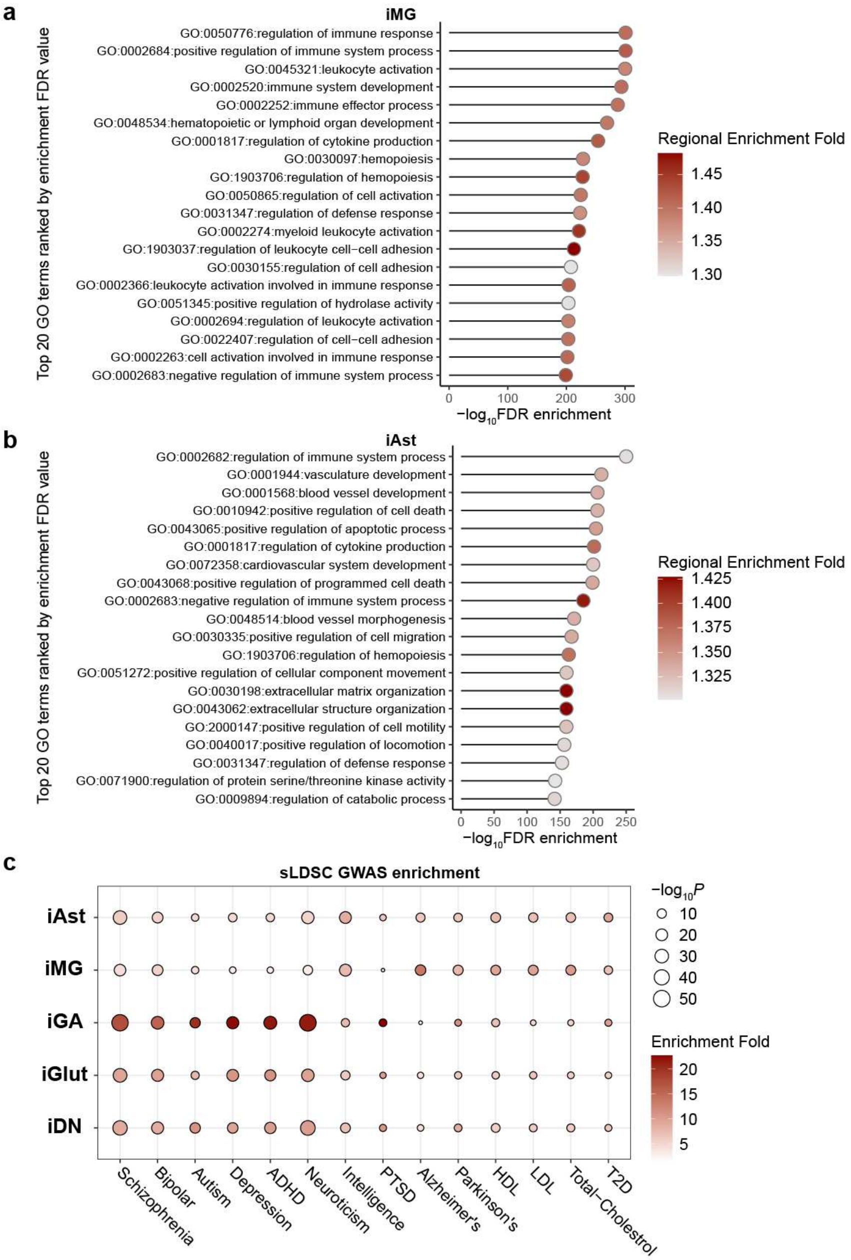


**Supplementary Fig. 1.** OCR peak enrichment for GO-terms and disease GWAS risk. (a) Top-ranking GO-terms enriched in iMG peaks. (b) Top-ranking GO-terms enriched in iAst peaks. (c) sLDSC enrichment analysis of OCR peaks for GWAS risk variants of LOAD and other NPD and

complex traits. Note the strongest enrichment of iMG peaks for LOAD GWAS risk. Disease GWAS datasets, Extended Data Table 8.

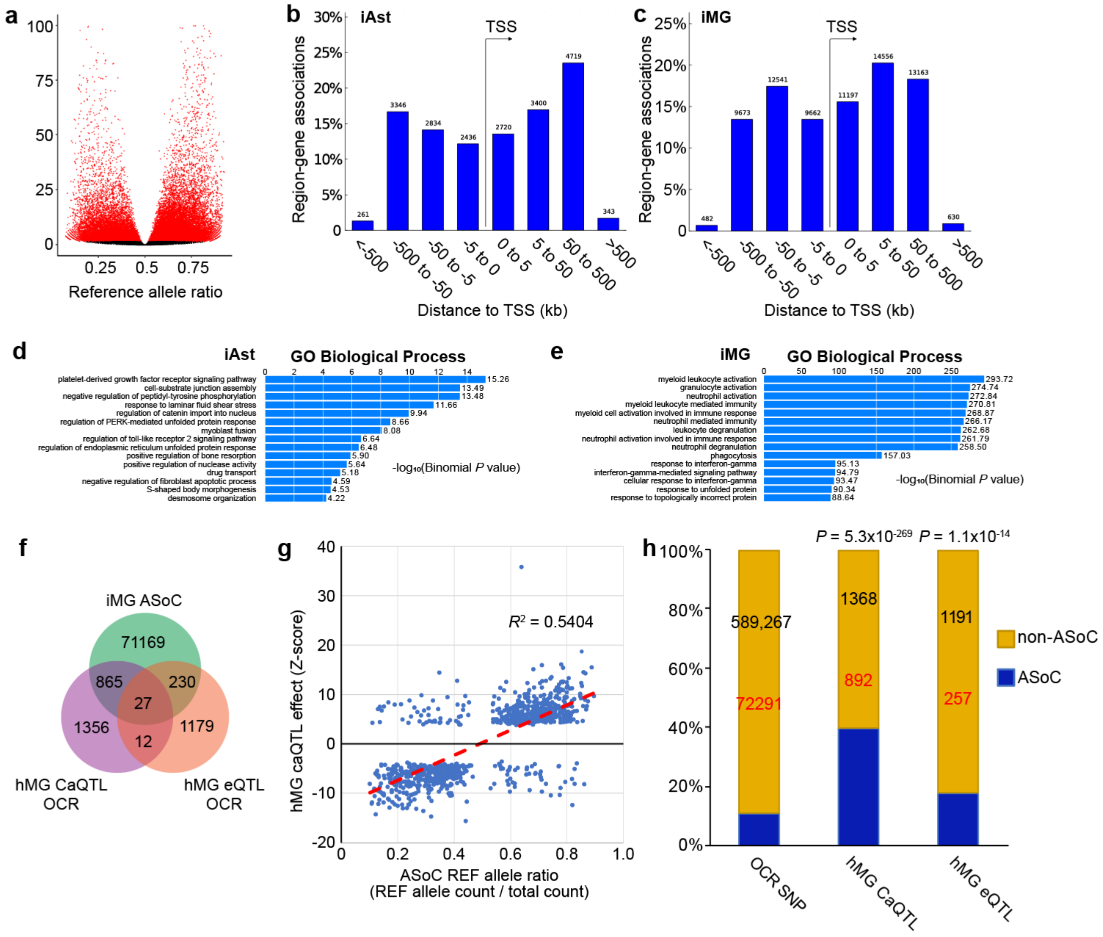


**Supplementary Fig. 2.** ASoC SNP characterization in iMG and iAst. (a) Example volcano plot of the reference allelic ratios of ASoC SNPs in iMG. SNPs showing ASoC (FDR<0.05) are in red. The binomial test was used to test ASoC by comparing the ATAC-seq read counts of the two alleles of an SNP for all heterozygous samples (see Method). (b) and (c) Distribution of the distances between ASoC SNPs and TSS of their nearest genes in iMG and iAst, respectively. (d) and (e) GO-term (biological processes) enrichment of the nearest genes of ASoC SNPs in iMG and iAst, respectively. Top-ranking GO-terms are shown. (b) to (e) were output from the GREAT analysis. (f) Venn diagram showing the overlap of iMG ASoC SNPs and previously reported hMG-caQTL and hMG-eQTL SNPs [18]. (g) Strong correlation of the reference allelic ratios of iMG ASoC SNPs and the allelic effect sizes of hMG-caQTL SNPs (n=892 overlapping SNPs). (h) The

percentage of brain hMG-caQRL and hMG-eQTL SNPs is also iMG ASoC SNPs (blue). The enrichment *P*-value of the overlapping ASoC SNPs (Fisher's exact test compared to the percentage of all ASoC SNPs in iMG OCRs) is listed at the top of each bar.

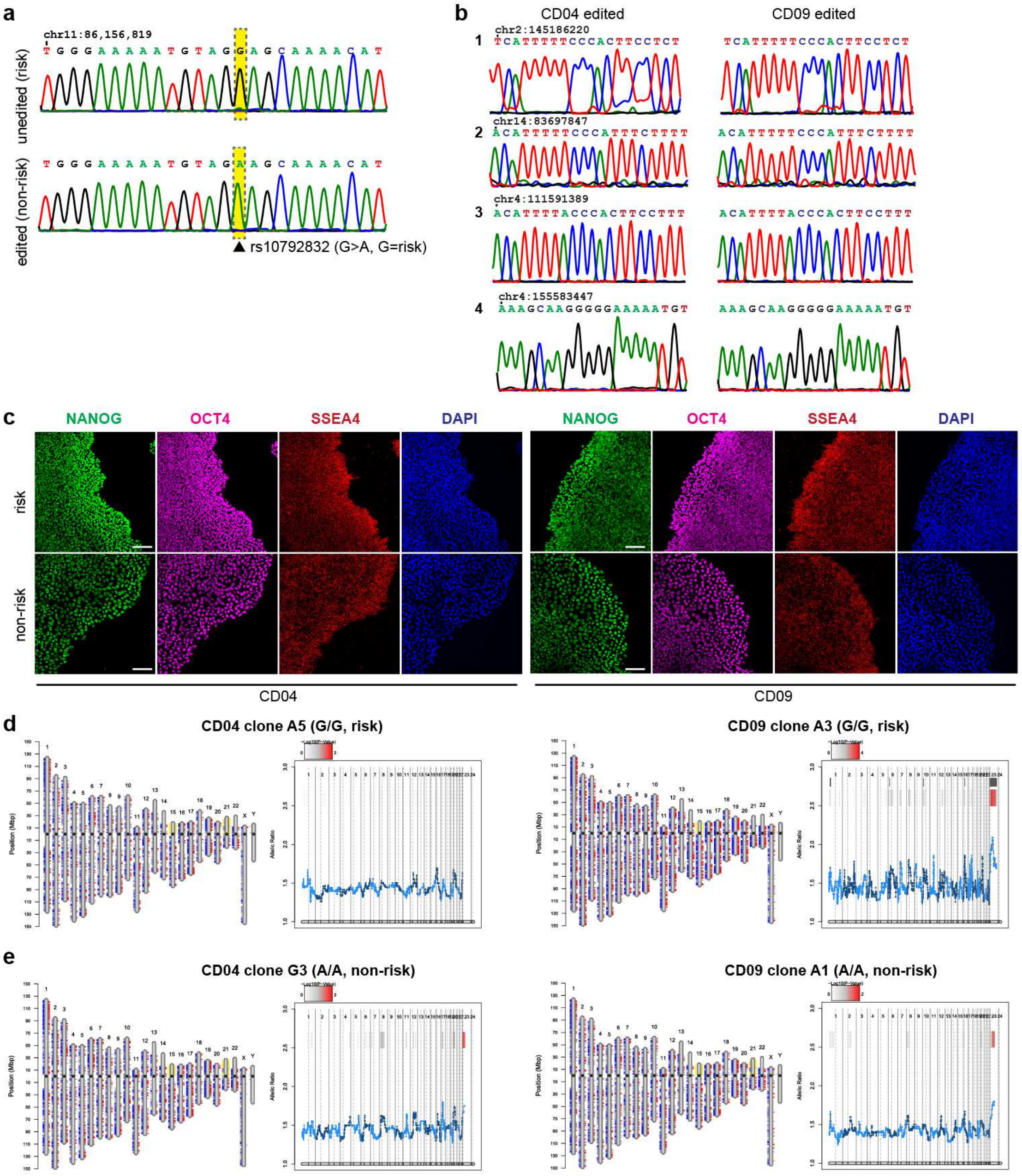


**Supplementary Fig. 3.** Characterization of CRISPR/Cas9 editing of rs10792832 in iPSC lines. (a) Sanger sequencing confirmation of the allele change of rs10792832 from G/G (unedited) to A/A (after editing) in iPSC. (b) Sanger sequencing confirmation of the absence of off-target editing. Representative sequencing traces are shown. (c) IF staining of stem cell pluripotency markers for the edited and unedited iPSC. Shown are representative images. (d) and (e) SNP e-

karyotyping for the two isogenic pairs of CRISPR-edited lines (CD04 and CD09) that carry G/G or A/A alleles. Note the RNA-seq data used for e-karyotyping were from iMG. For each iPSC clone used for iMG differentiation, the left panel is the chromosomal view of SNP heterozygosity, and the right panel is a moving average of SNP allelic ratios across the transcribed genome. No obvious chromosomal abnormality was observed.

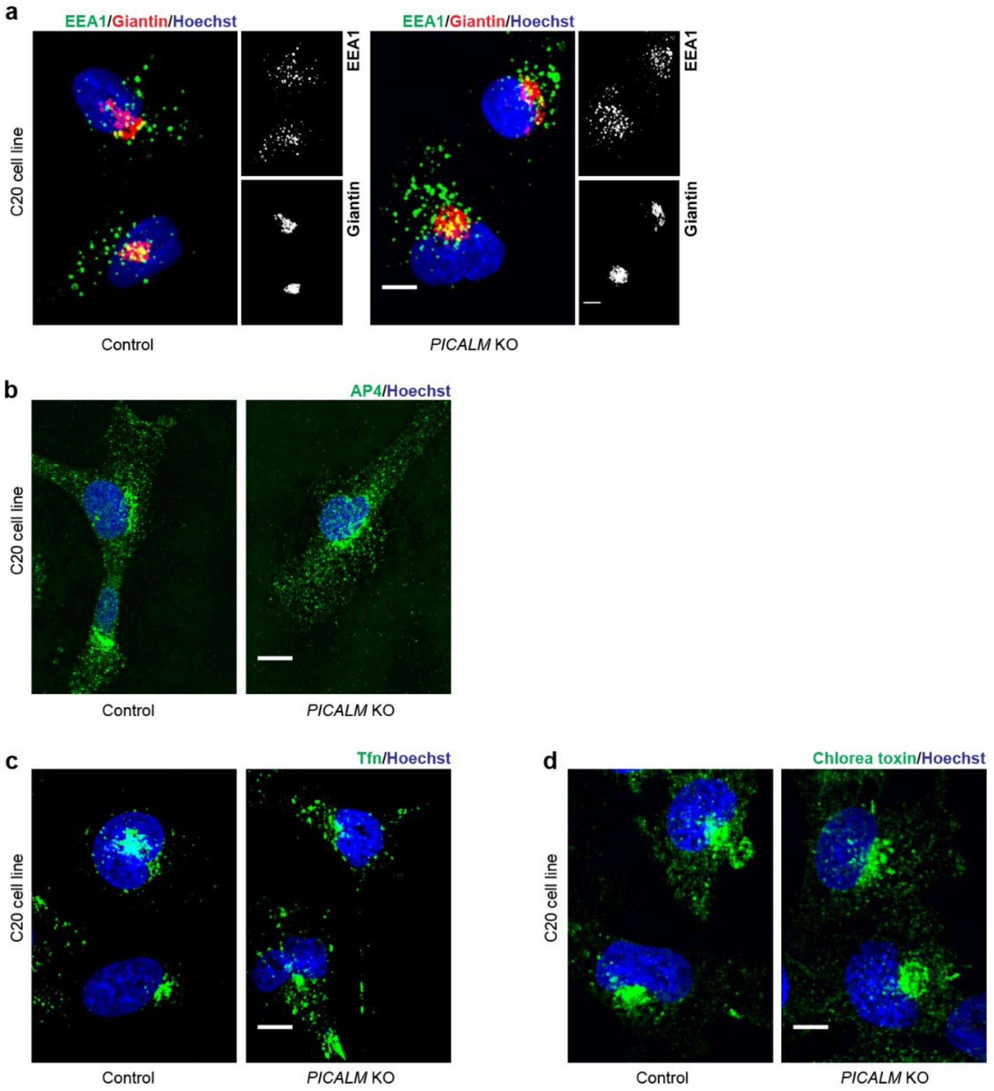


**Supplementary Fig. 4**. Basic functional organelle characterization of C20 PICALM-KO cells. (a) IF staining of EEA1 and Giantin indicates normal early endosomes and Golgi apparatus in KO cells. (b) IF staining of AP4 shows normal endocytic vesicles in KO cells. (c) and (d) IF staining of Tfn uptake and cholera toxin shows normal endocytic flow in KO cells. Note that endocytosis of Tfn is through clathrin-dependent endocytosis, while endocytosis of CTx is through clathrin-dependent and clathrin-independent pathways and caveolae-mediated internalization.

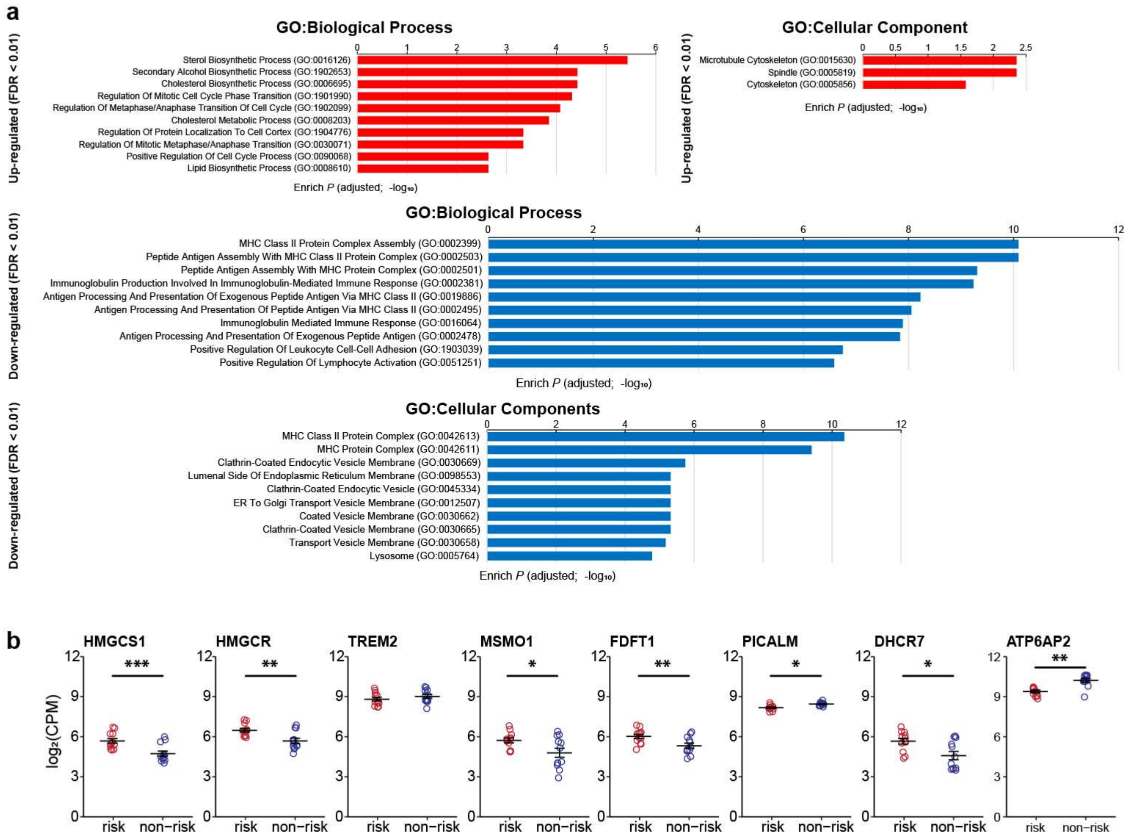


**Supplementary Fig. 5**. DE analysis of iMG carrying the LOAD risk allele of *PICALM* and GO-term enrichment analysis. (a) GO-term (enrichment analysis of top-ranking up-regulated (FDR<0.01) genes (top panels) and down-regulated (FDR<0.01) genes (bottom panels). EnrichR was used for enrichment analysis [71]. Only the top 10 enriched GO-terms are listed (biological process, left; cellular component, right). (b) The expression difference of the selected genes. Each dot represents each RNA-seq sample. CPM, count per million. Adjusted *P*-values are from DE analysis. (c) Venn diagram Overlapping DE genes (FDR<0.05) in iMG carrying the LOAD risk allele of *PICALM* (vs. non-risk) and in mouse MG with high LD (vs. low LD). (d) Pearson's correlation of the expression changes of the 73 overlapping genes between the two DE gene lists in (c). Of the 73 overlapping DE genes, 45 show the same directional DE changes, two-tailed Fisher's exact *t*-test, *P* < 0.013.
